# Chronic Probing of Deep Brain Neuronal Activity Using Nanofibrous Smart Conducting Hydrogel‐Based Brain–Machine Interface Probes

**DOI:** 10.1002/smsc.202400463

**Published:** 2025-01-28

**Authors:** Seyed Shahrooz Zargarian, Chiara Rinoldi, Yasamin Ziai, Anna Zakrzewska, Roberto Fiorelli, Małgorzata Gazińska, Martina Marinelli, Magdalena Majkowska, Paweł Hottowy, Bartosz Mindur, Rafał Czajkowski, Ewa Kublik, Paweł Nakielski, Massimiliano Lanzi, Leszek Kaczmarek, Filippo Pierini

**Affiliations:** ^1^ Department of Biosystems and Soft Matter Institute of Fundamental Technological Research Polish Academy of Sciences 02‐106 Warsaw Poland; ^2^ Department of Engineering and Technology of Polymers Faculty of Chemistry Wrocław University of Science and Technology Wyb. Wyspiańskiego 27 50‐370 Wroclaw Poland; ^3^ Department of Industrial Chemistry “Toso Montanari” Alma Mater Studiorum University of Bologna 40136 Bologna Italy; ^4^ BRAINCITY Nencki Institute of Experimental Biology Polish Academy of Sciences 02‐093 Warsaw Poland; ^5^ Faculty of Physics and Applied Computer Science AGH University of Krakow 30‐059 Krakow Poland; ^6^ Center for Basic and Translational Research in Biology and Biomedical Sciences Nencki Institute of Experimental Biology Polish Academy of Sciences 02‐093 Warsaw Poland

**Keywords:** brain–machine interfaces, conducting nanofibrous hydrogels, electrophysiologies, neural activity recordings, semi‐interpenetrating polymer networks

## Abstract

The mechanical mismatch between microelectrode of brain–machine interfaces (BMIs) and soft brain tissue during electrophysiological investigations leads to inflammation, glial scarring, and compromising performance. Herein, a nanostructured, stimuli‐responsive, conductive, and semi‐interpenetrating polymer network hydrogel‐based coated BMIs probe is introduced. The system interface is composed of a cross‐linkable poly(*N*‐isopropylacrylamide)‐based copolymer and regioregular poly[3‐(6‐methoxyhexyl)thiophene] fabricated via electrospinning and integrated into a neural probe. The coating's nanofibrous architecture offers a rapid swelling response and faster shape recovery compared to bulk hydrogels. Moreover, the smart coating becomes more conductive at physiological temperatures, which improves signal transmission efficiency and enhances its stability during chronic use. Indeed, detecting acute neuronal deep brain signals in a mouse model demonstrates that the developed probe can record high‐quality signals and action potentials, favorably modulating impedance and capacitance. Evaluation of in vivo neuronal activity and biocompatibility in chronic configuration shows the successful recording of deep brain signals and a lack of substantial inflammatory response in the long‐term. The development of conducting fibrous hydrogel bio‐interface demonstrates its potential to overcome the limitations of current neural probes, highlighting its promising properties as a candidate for long‐term, high‐quality detection of neuronal activities for deep brain applications such as BMIs.

## Graphical abstract

Herein, nanofibrous smart conducting hydrogel‐based brain–machine interface probe that overcomes mechanical mismatches in deep brain electrophysiology is introduced. Herein, through this novel probe, signal quality and stability are enhanced, inflammation is reduced, and long‐term recordings are supported. In in vivo mouse model tests, high‐quality deep brain signal recording and minimal inflammatory response are demonstrated, highlighting its potential for chronic brain–machine interface applications.

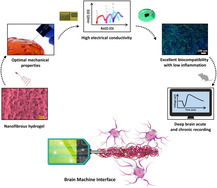

## 1 Introduction

Electrophysiological investigations, specifically those requiring intracranial electrodes, hold immense promise for revolutionizing the treatment of neurological disorders and injuries.^[^ ^1^, ^2^, ^3^ ^]^ By enabling direct communication between the brain and external devices, neural probes offer ground‐breaking possibilities for restoring lost functions, controlling prosthetics, and even enhancing human capabilities. The main clinical applications of electrophysiology in human medicine are deep brain stimulation (DBS), brain–machine interface (BMI), and stereo‐electroencephalography (sEEG). DBS is a well‐established technique,^[^ ^4^ ^]^ that uses electrical impulses to modulate the activity of diseased brain tissue (e.g., for treating Parkinson's disease). Two others use electrodes to record neuronal signals—sEEG looks for epileptogenic focus in deep brain areas; in BMI, the signal recorded from the motor cortex of a paralyzed person is translated to commands directing the “machine” (i.e., computer or computer‐controlled prosthesis or robot).

While advancements in microelectrode technology have yielded impressive capabilities, a critical challenge in deep brain electrophysiology remains in achieving long‐term functionality: the mechanical mismatch between rigid BMI's neural probe and the soft brain tissue.^[^ ^5^ ^]^ Overtime, during chronic implantation, this challenge leads to reduced tissue integration, increased inflammation, formation of glial scars, and, ultimately, reduction in signal acquisition or stimulation quality.^[^ ^6^ ^]^ Synchronizing micromotions of the probe with the brain tissue fosters a stable interface, potentially mitigating inflammation and preventing glial scar formation. This target can be relatively easily achieved with superficial, “floating” cortical electrodes but is very challenging in the case of deep brain recording with long, rigid probes.^[^ ^7^, ^8^ ^]^ As a potential solution, researchers have explored using soft coatings at the neural probe's surface to address this issue.

Soft coatings add flexibility and compatibility to the microelectrode, promoting better tissue integration.^[^ ^9^ ^]^ Numerous studies have been directed toward fine‐tuning their electrical and mechanical properties.^[^ ^10^, ^11^ ^]^ The primary goal is to develop soft and pliable conducting coatings that closely mimic the electrochemical and mechanical characteristics of living neural tissue. These coatings must exhibit high biocompatibility, excellent wettability, and appropriate electrical properties.^[^ ^12^ ^]^ Polymeric hydrogels have proven to be highly practical for this purpose, satisfying the first two requirements with ease. Notably, Young's modulus of these soft materials is three orders of magnitude lower than that of most commonly used biomaterials in the field of neural interfaces.^[^ ^13^ ^]^ Furthermore, polymeric hydrogels promote tissue integration, with their high swelling characteristic firmly integrating with the neural probe in brain tissue and enhancing adaptation to the brain's contoured surfaces.^[^ ^14^ ^]^ Unfortunately, the electrical properties of hydrogels still need significant improvements to achieve optimal recording resolution. It is worth stressing that the priority for a BMI material in human applications is biocompatibility without the loss of signal quality; the expectation of enhanced signal power is excessive and should not be considered a necessary requirement for such materials. Incorporating a conducting material into the coating fulfills the third requirement for neural prosthetic applications, i.e., appropriate electrical properties.^[^ ^10^ ^]^ Intrinsically conducting polymers (ICPs) have received considerable attention due to their excellent performance and safety in physiologic environments.^[^ ^15^ ^]^ However, the relatively high rigidity of ICPs poses a challenge to the mechanical mismatch between the neural probe surface and the brain tissue.

While neither polymeric hydrogel nor ICPs alone provide a stable performing interface between an implant and the soft brain tissue, their combination may benefit from enhanced mechanical and electrochemical properties.^[^ ^16^ ^]^ In this sense, multicomponent conducting polymer hydrogels (CPHs) can be utilized as a conducting and soft interface with the neural tissue. The addition of the conducting component can coincide with the polymerization or the cross‐linking process of the latter. As a result, a semi‐interpenetrating polymer network (semi‐IPN) can be formed, which benefits from a synergistic increase in electrical and mechanical properties compared to its blended form.

Despite recent progress, further efforts are necessary to increase the long‐term stability of the soft conducting coatings for in vivo applications. For instance, the poor adhesion of a hydrogel layer to the substrate limits the microelectrode operability. More concern is that applied concentrations of the conducting polymers may cause a noticeable discrepancy between the mechanical properties of the CPHs and the brain tissue, consequently decreasing the stability of the hydrogel coating due to the lower water content.

The miniaturization of neural‐probe‐coating structures, enabled by techniques like electrospinning, offers a promising solution to address the challenges associated with conventional hydrogel coatings.^[^ ^17^, ^18^ ^]^ Electrospinning allows for the fabrication of highly intricate, nanofibrous architectures that closely mimic the structural and mechanical properties of the extracellular matrix.^[^ ^19^, ^20^ ^]^ These nanofibrous structures offer significant improvements in mechanical compliance and integration with neural tissue. The increased surface area and porosity of these coatings facilitate rapid water uptake and swelling, which are necessary for maintaining a stable and conductive interface with the surrounding tissue. Furthermore, electrospinning facilitates the incorporation of an ICP into a nanostructured stimuli‐responsive hydrogel that enhances the electrical properties of the BMI's microelectrode coating, making it more responsive and suitable for long‐term, chronic applications. This level of control and precision, challenging to achieve with bulk hydrogels, shows the innovative potential of smart nanostructured coatings in advancing neural interface technology.

To this end, we aim to develop a nanostructured smart conducting hydrogel‐based coated BMI probe for electrophysiological investigations. We focused on poly(*N*‐isopropylacrylamide) (PNIPAM) for the coating component. This thermoresponsive polymer has garnered significant attention due to its reversible volume phase transition (VPT) at the lower critical solution temperature (LCST).^[^ ^21^ ^]^ We equipped the PNIPAM polymeric chains with cross‐linkable groups. With its highly porous structure and ability to exhibit a high swelling ratio, our PNIPAM‐based hydrogel presents an efficient 3D matrix for the formation of the semi‐IPN. Concerning the conducting component, we made our selection from the polythiophene (PT) family. The high conductivity of PT comes from synthesis techniques that promote regioregular head‐to‐tail (HT) coupling and increased orbital overlap along the polymer backbone.^[^ ^22^, ^23^ ^]^ A diverse range of functional end‐groups can also be incorporated into PT.^[^ ^24^ ^]^ We took advantage of this property and chemically tailored our PT's polymeric chains to suit the targeted application. The miniaturization of the PNIPAM copolymer and modified PT (regioregular poly[3‐(6‐methoxyhexyl)thiophene] [PT6OMe]) was performed via electrospinning. The construct was later turned into a semi‐IPN conducting fibrous hydrogel via a mild cross‐linking reaction. A complete analysis was directed at the semi‐IPN's physical structure, chemical composition, electrical property, and mechanical performance. The fibrous semi‐IPN was then weaved around the neural probe shaft. This approach aims to achieve several key improvements: rapid swelling response, high water uptake, and enhanced adhesion to the neural probe shaft, particularly at physiological temperatures. Additionally, the design strategy seeks to overcome the trade‐off between achieving optimal conductivity and maintaining strong mechanical properties within the nanostructured semi‐IPN. This could potentially lead to a lower impedance during implantation procedures. Furthermore, the biological responses of L929 fibroblast cells in indirect and direct contact with the fibrous semi‐IPN were assessed in terms of cell viability and morphology. Finally, in vivo implantation of neural probes coated with the nanostructured semi‐IPN was performed on a mouse model. Performance evaluations were assessed by means of electrophysiology and quality of recordings at acute and chronic phases in the thalamus, while chronic inflammatory responses were investigated by histological analysis.

## 2 Results and Discussion

**Figure 1:**
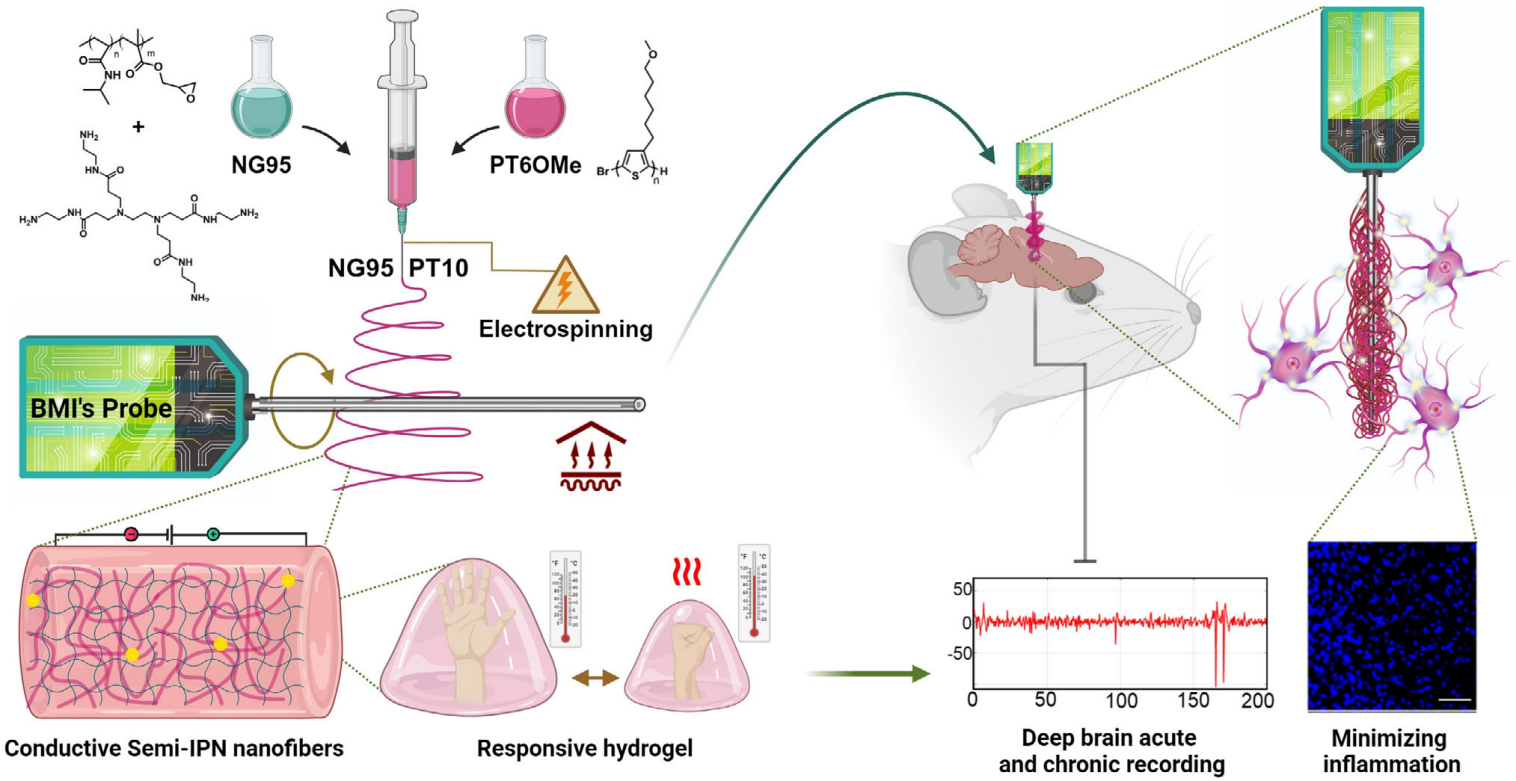
Schematic illustration of the BMI probe fabrication, structure, and final application in vivo. Top left: fabrication process in which the cross‐linker is mixed with the synthesized temperature‐responsive copolymer and PT6OMe. The mixture is then electrospun, and the resulting nanofibers are collected on a BMI probe. Bottom left: a magnified view of the conductive semi‐IPN fibrous hydrogel coating revealing its temperature‐responsive behavior. Right: the in vivo application depicts the coated BMI probe inserted into a mouse brain, enabling deep brain acute and chronic recordings while minimizing inflammation.

A biocompatible soft material with low impedance is required to create a suitable coating for a neural probe. This coating must effectively adhere to the probe's surface during recording or stimulation, maintain mechanical compatibility with the brain for a stable connection, and offer low impedance for efficient electrical signal transmission from neurons to the probe (**Figure** **1**). In this study, we present the synthesis and production of a nanostructured semi‐IPN hydrogel designed to fulfill these requirements as a coating for neural probes.

### 2.1 Synthesis of 2,5‐Dibromo‐3‐(6‐Methoxyhexyl)thiophene and Regioregular PT6OMe

**Figure 2:**
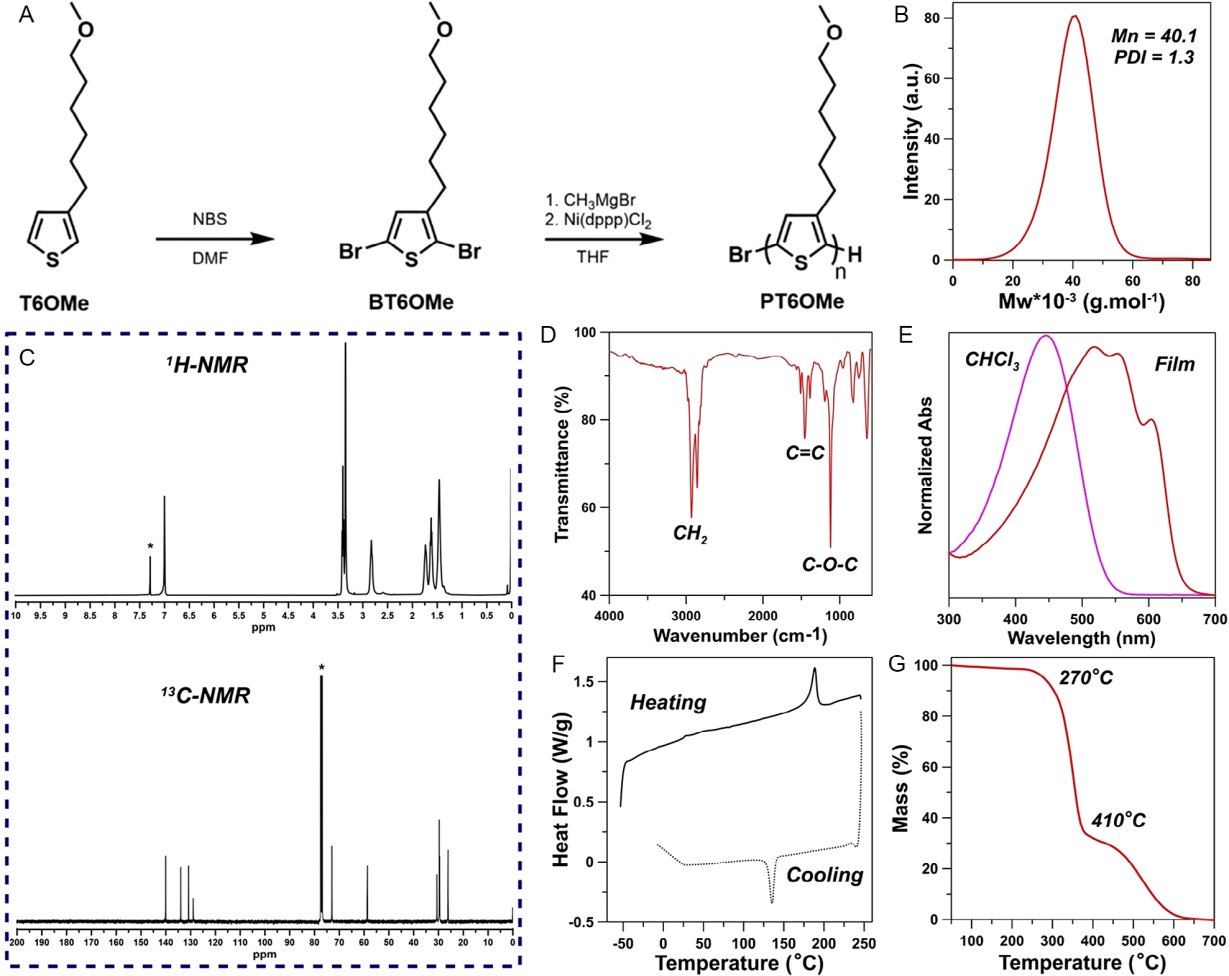
Chemical synthesis and characterization of the PT6OMe. A) Synthetic pathway to obtain the desired polymer PT6OMe and its B) GPC eluogram; C) ^1^H‐NMR and ^13^C‐NMR spectra; D) FT‐IR spectrum; E) UV–Vis spectra in CHCl_3_ solution and thin film; F) DSC curves; and G) TGA thermogram in air.

Synthesis of a regioregular PT with enhanced electronic properties for effective transmission of neural signals has been carried out. The starting thiophenic monomer 2,5‐dibromo‐3‐(6‐methoxyhexyl)thiophene (BT6OMe) was polymerized using the procedure described by McCullough et al.^[^ ^25^ ^]^ This method, involving a magnesium–halogen exchange (metathesis reaction) with a preformed aliphatic Grignard derivative (methylmagnesium bromide) followed by a Ni(II)‐catalyzed cross‐coupling step, directly gave the regioregular polymer PT6OMe with a high yield (77%) (**Figure** **2**a), good molecular weight (40.1 kDa), and polydispersity index (1.28) (Figure 2b). The presence of a singlet at 6.97 ppm in the region of aromatic protons of the ^1^H‐NMR spectrum of the polymer, as well as the appearance of only four signals ascribable to the four aromatic carbons in ^13^C‐NMR spectrum (Figure 2c, S1, Supporting Information, and Experimental Section) clearly evidences the high degree of regioregularity of PT6OMe (98% HT). The chemical identity of the polymer is also confirmed by the fourier transform infrared spectroscopy (FT‐IR) spectrum (Figure 2d) and elemental analysis (Experimental Section). These findings comply with the expected structure of the synthesized material.

UV–Vis spectra of PT6OMe in chloroform solution and in film on a quartz slide are shown in Figure 2e, where the two curves are normalized to their maximum intensity. The solution spectrum shows a *λ*_max_ at 456 nm, while that of polymer film shows a *λ*_max_ at 521 nm and two shoulders at 558 and 603 nm, corresponding to the second and the first vibronic quantum, respectively. The remarkable bathochromic shift of 65 nm from a single structureless band of π−π* origin (centered at 456 nm) to a structured one with *λ*_max_ = 521 nm, observed when passing from solution to film, is indicative of the conformational self‐assembling capability of the polymer chains when examined in the solid state.^[^ ^26^ ^]^ The transition from solution to film involves the doubling of the mean conjugation length of the polythiophenic chromophore, from 7 to 14 conjugated thiophenic rings,^[^ ^27^ ^]^ suggesting that the methoxy‐functionalized hexylic side chain plays an important role in controlling polymer chains mobility and rotational freedom, hence leading to the prevalence of more planar and conjugated backbone conformations when in the solid state.

The differential scanning calorimetry (DSC) thermogram of PT6OMe (Figure 2f) shows an endothermic flexure at 31 °C (glass transition temperature, *T*_g_) and also an endothermic peak at 173 °C (melting of crystalline domains) in the heating run and only an exothermic peak at 134 °C during the cooling step. Polymer thermal stability was investigated by the thermogravimetric analysis (TGA) in air, and the thermogram is reported in Figure 2g. The decomposition starts at about 270 °C (loss of side chains) and a second step, involving the polymeric backbone, at about 410 °C. To study the molecular arrangement of PT6OMe film, the X‐ray diffraction (XRD) pattern was recorded and is shown in Figure S2, Supporting Information. The film examined showed three characteristic peaks at the small angles assigned to (100), (200), and (300) reflections, respectively (Table S1, Supporting Information). The first‐order reflection (100) may be ascribed to the distance between the polythiophenic chains on the same plane, while multiple X‐ray reflections by the film induce (200) and (300) peaks. The observed *d*‐spacing (16.21 Å), corresponding to the distance between the thiophene molecules belonging to the same plane, is shorter with respect to the double of the calculated side chain length (18.42 Å), as determined by the density‐functional theory (DFT) for the B3LYP/6‐31G(d) level. This indicates that side chains are partially interdigitated, providing a 3D chain ordering that can result in enhanced charge‐transport properties.^[^ ^28^ ^]^ Moreover, the less evident peak at wide angles (2*θ* = 21.80°), partially embedded within the broad, amorphous halo, and ascribable to the presence of *π−π* stacking of the thiophene rings in the main chains, confirms the semicrystalline nature of the examined polymer. Finally, the mean size of polymer crystallites (*L*), evaluated using Scherrer's equation (Table S1, Supporting Information), indicates that the polymer is characterized by quite extended crystalline domains (15.95 nm).^[^ ^29^ ^]^ Figure S3, Supporting Information, reports the current–voltage (I–V) curves measured in a vacuum (10^−3^ mmHg) and in an ambient atmosphere. The fitting curves show satisfying symmetry and linearity throughout the measured range (*R* ^2^ = 0.9995 in vacuum and 0.9991 in air), thus confirming the resistor‐like behavior of the polymeric film. Electrical conductivity was 2.44 × 10^−4^ S cm^−2^ in vacuum and 2.80 × 10^−4^ S cm^−2^ in air, and the slightly higher value obtained under ambient atmosphere could be explained in terms of partial oxygen doping of the polymer.^[^ ^30^ ^]^

### 2.2 Copolymerization of P(NIPAM‐co‐GMA) and Fabrication of Bulk Hydrogel

**Figure 3:**
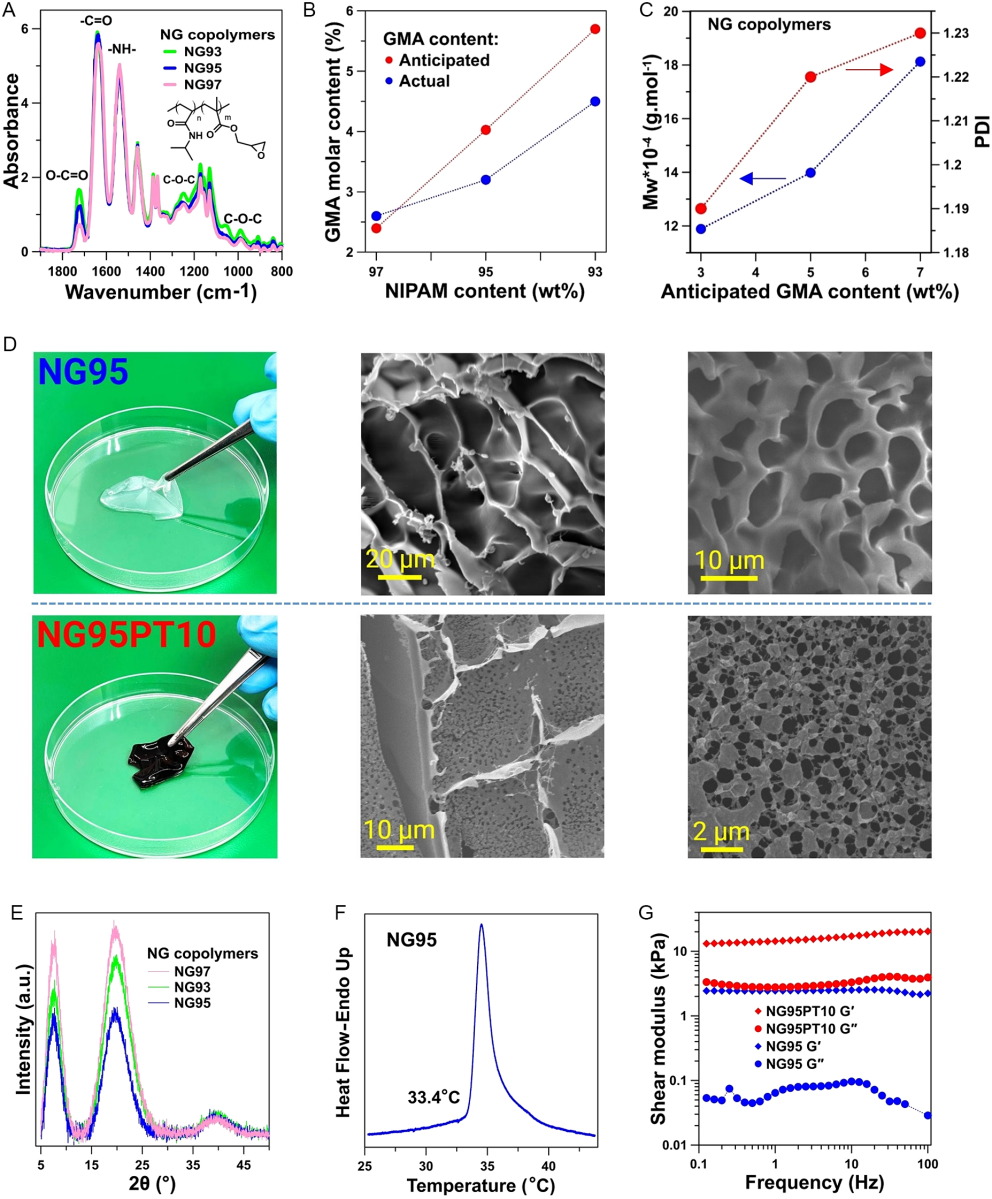
P(NIPAM*‐co*‐GMA) synthesis, cross‐linking, and characterization. A) FT‐IR of the synthesized copolymers. B) Discrepancy track of GMA molar content. C) Mw and PDI of NG copolymers. D) NG95 and NG95PT10 bulk hydrogels: camera image and SEM micrographs at different magnifications for each hydrogel (NG95 upper row, NG95PT10 lower row). E) XRD of NG copolymers. F) Assessing the LCST of NG95 aqueous solution. G) DMA analysis NG95PT10 and NG95 bulk hydrogels.

In the next phase, we synthesized a stimuli‐responsive cross‐linkable polymer derived from *N,N*‐isopropylacrylamide (NIPAM). This polymer was designed to function as the soft component of the neural probe's coating, possessing mechanical properties that align with those of brain tissue. An ammonium persulfate (APS)‐initiated *N,N,N′,N′*‐tetramethylethylenediamine (TEMED)‐accelerated copolymerization of pristine monomers of NIPAM and glycidyl methacrylate (GMA) in an aqueous media was conducted (Figure S4, Supporting Information). The FT‐IR spectra of the dialyzed copolymers are presented in **Figure** **3**a. As the proportion of NIPAM monomers in the initial feed decreases (from P(NIPAM‐co‐GMA) [NG]97, with an NIPAM/GMA feed ratio of 97/3, to NG93, where this ratio is 93/7), the characteristic peaks of GMA at 1720, 1240, and 910 cm^−1^ exhibit progressive increases. These peaks correspond to the O—C=O stretching of GMA's ester configuration and the C—O—C stretching of epoxide groups, respectively. The in‐chain molar fractions of the NIPAM and GMA units were determined by the nuclear magnetic resonance (NMR) measurement (Figure S5, Supporting Information, and further detailed in Figure S6, Supporting Information) and compared to that of the initial feed. The compositions of the resulting NG copolymers are plotted in Figure 3b. As seen, the actual GMA content in NG97 is higher than its anticipated molar proportion in the initial feed. When the molecular weight and polydispersity are considered constant, the discrepancy in the mentioned values represents the high reactivity of one monomer over the other. We observed a surge in the molecular weight of the copolymers having higher GMA content in the feed (Figure 3c and Table S2, Supporting Information). The gel permeation chromatography (GPC) curves were of single modal, and all the copolymers had high molecular weight. The high molecular weight of the copolymers (118 000 < Mw < 230 000 Da) and their narrow distribution (1.14 < polidispersity index (PDI) < 1.23) take a pivotal role during the electrospinning process. The slightly hydrophobic nature of NG93, on the one hand, and the low amount of functional epoxy groups in NG97, on the other hand, resolved the selection of NG95 for the main experiments in this study. To create a 3D structure using NG, we opted for a cytocompatible cross‐linker, poly(amidoamine) (PAMAM) dendrimer, which has four primary amine groups. The cross‐linking reaction is schematically represented in Figure S7, Supporting Information. The bulk hydrogels of NG95 and NG95PT10 (with 10% w/w of PT6OMe) are represented in Figure 3d. Both bulk hydrogels exhibited a 3D structure with two groups of macropores having a relatively uniform distribution. The interconnected porous cell‐like structure of NG95 remained intact upon the introduction of PT. This suggests that the presence of the conductive counterpart in the semi‐IPN bulk hydrogel did not disrupt the cross‐linking process of NG95. This observation was beneficial as with having the ultimate goal of electrospinning and cross‐linking of NG95, the amount of PAMAM and PT6OMe should be carefully considered. Crystalline phases in NG95 were assessed using XRD and compared to their counterparts, as depicted in Figure 3e. Generally, NG with varying monomer ratios exhibits two distinct diffraction peaks associated with the semicrystalline structure of PNIPAM's soft and hard segments. The broad, diffuse diffraction peak at 2*θ* = 20° stems from intermolecular interactions among nonbonded atoms, while the peak at 2*θ* = 8.5° arises from interpolymer chain correlations attributed to their bulky side groups.^[^ ^31^ ^]^ Moreover, the LCST of NG95 was detected at 33.4 °C, which is similar to that of pure PNIPAM (Figure 3f).^[^ ^21^ ^]^ The mechanical properties of NG95PT10 and NG95 hydrogels are shown in Figure 3g. At 1 Hz, the storage modulus of NG95PT10 (10.9 ± 0.14 kPa) is approximately four and a half times higher than that of NG95 hydrogel (2.4 ± 0.2 kPa). This extreme difference can be attributed to stiff PT6OMe polymeric chains inside the semi‐IPN structure since polythiophenic main chains can give inter‐ and intrachain π‐stacking interactions. Moreover, the loss modulus of NG95PT10 (2990 ± 104 Pa) is almost 44 times higher than that of the bare NG95 hydrogel (68 ± 25 Pa) at 1 Hz. Still, the mechanical properties of NG95PT10 hydrogel, in its standalone state, fall within the range of values reported for brain tissue,^[^ ^32^ ^]^ particularly at low to mid‐range frequencies,^[^ ^33^, ^34^ ^]^ indicating that the hydrogel itself is mechanically compatible with the viscoelastic properties of brain tissue. However, it is important to note that this compatibility pertains to the hydrogel alone and not the combined hydrogel‐coated BMI's probe. When used as a coating for a neural probe, the shear modulus of NG95PT10 hydrogel contributes to its ability to elastically respond to micro‐displacements in brain tissue.

### 2.3 Fabrication and Characterization of Nanostructured Hydrogels

**Figure 4:**
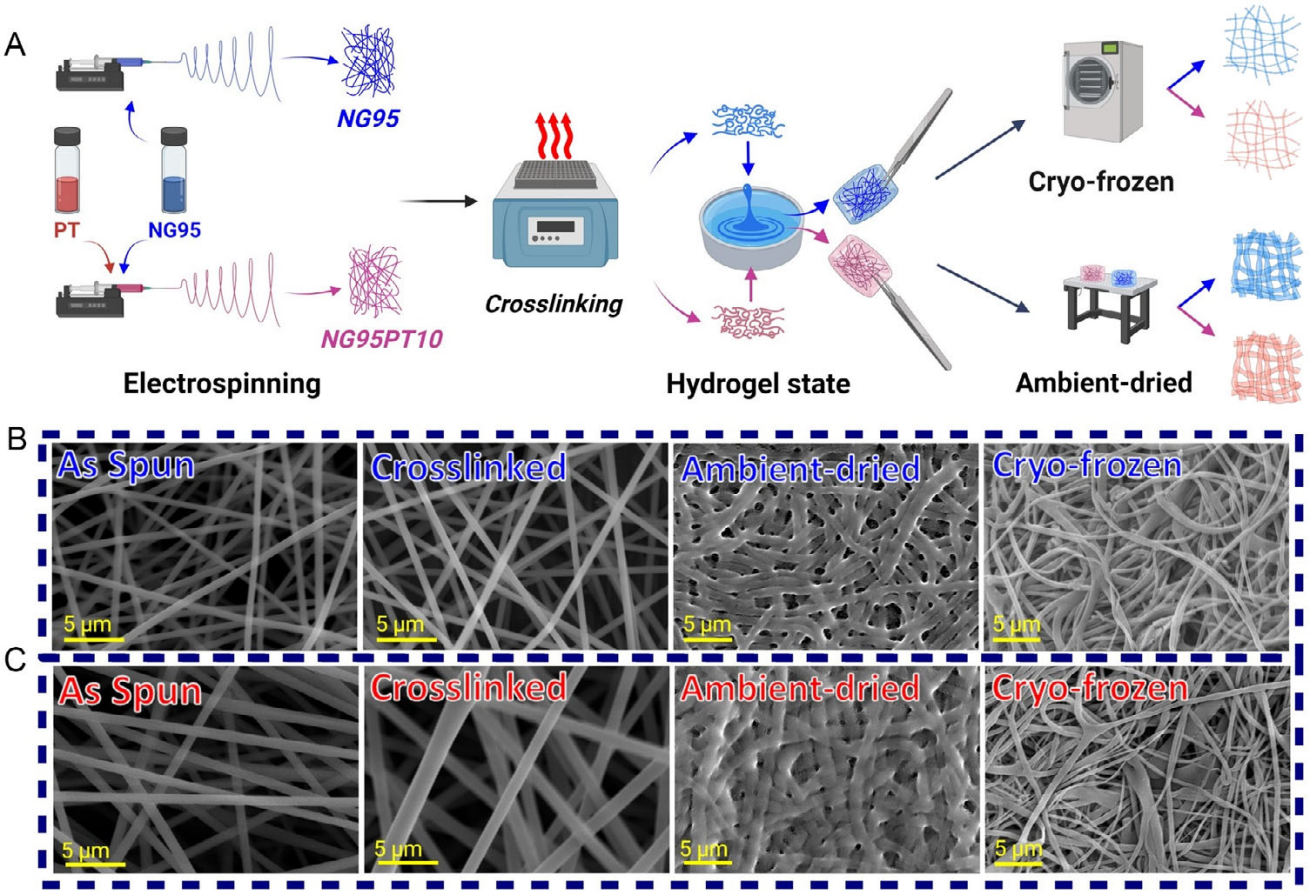
Fabrication of the semi‐IPN fibrous hydrogel. A) Schematic illustration of the electrospinning‐based fabrication process for NG95 and NG95PT10 fibrous hydrogels. B) SEM micrographs of as‐spun and cross‐linked NG95 nanofibers reveal a smooth, bead‐free morphology. Next, micrographs demonstrate a distinct difference in morphology between ambient‐dried and cryo‐frozen NG95 hydrogel fibers, with ambient‐dried fibers taking on a ribbonlike shape and cryo‐frozen fibers maintaining a circular form. C) SEM micrographs of NG95PT10 fibers show a slightly larger AFD compared to NG95 fibers. Similar observations were made regarding the drying condition effects on NG95PT10 hydrogel fibers.

The anisotropic structures produced by electrospinning have exceptionally higher porosity and specific surface area, lower thickness, and better mechanical properties. However, this fabrication technique cannot be used to electrospin hydrogels. This study developed electrospinning of hydrophilic cross‐linkable copolymers, followed by post‐electrospinning heat treatments, as an alternative route. The mixture of NG95 and PAMAM dendrimers exhibited excellent electrospinability when dissolved in a combination solvent system of chloroform and *N,N*‐dimethylformamide (DMF). Consequently, a defect‐free fibrous mat was successfully obtained. The functional epoxy groups derived from GMA form the backbone of the copolymer's on‐demand cross‐linking capability (Figure S8, Supporting Information). The cross‐linking of the fibrous structure occurred through a condensation reaction at 53 °C, with the optimal cross‐linking time being 120 min. Afterward, both NG95 and NG95PT10 were converted into their hydrogel state. Two methods were employed to study their morphology: one involved allowing them to dry under ambient conditions, and the other included cryo‐freezing the samples. These steps are schematically illustrated in **Figure** **4**a. The morphology of the cross‐linked fibrous membrane was examined using scanning electron microscope (SEM, Figure 4b,c). Heat treatment induced a noticeable reduction in the size of NG95 fibers, leading to a decrease in the average fiber diameter (AFD). Following the cross‐linking process, the AFD of NG95 decreased to 540 nm, displaying a single‐modal distribution. Interestingly, NG95PT10 fibers did not undergo a significant change in AFD after cross‐linking, primarily due to the less hydrophilic nature of PT6OMe. Notably, the drying method significantly impacted the morphology of the electrospun fibers. Air‐drying resulted in ribbonlike fibers, likely due to the gravitational pull acting along the length of the fibers as the water evaporated. Conversely, cryo‐drying using a freeze‐drying technique yielded fibers with a reduced diameter compared to their swollen state. This phenomenon can be attributed to the uniform pressure exerted by the vacuum environment during the sublimation of the water molecules. Furthermore, the formation of fibrous hydrogels was found to be reproducible across multiple hydration and dehydration cycles, although it had an irreversible impact on fiber diameter.

**Figure 5:**
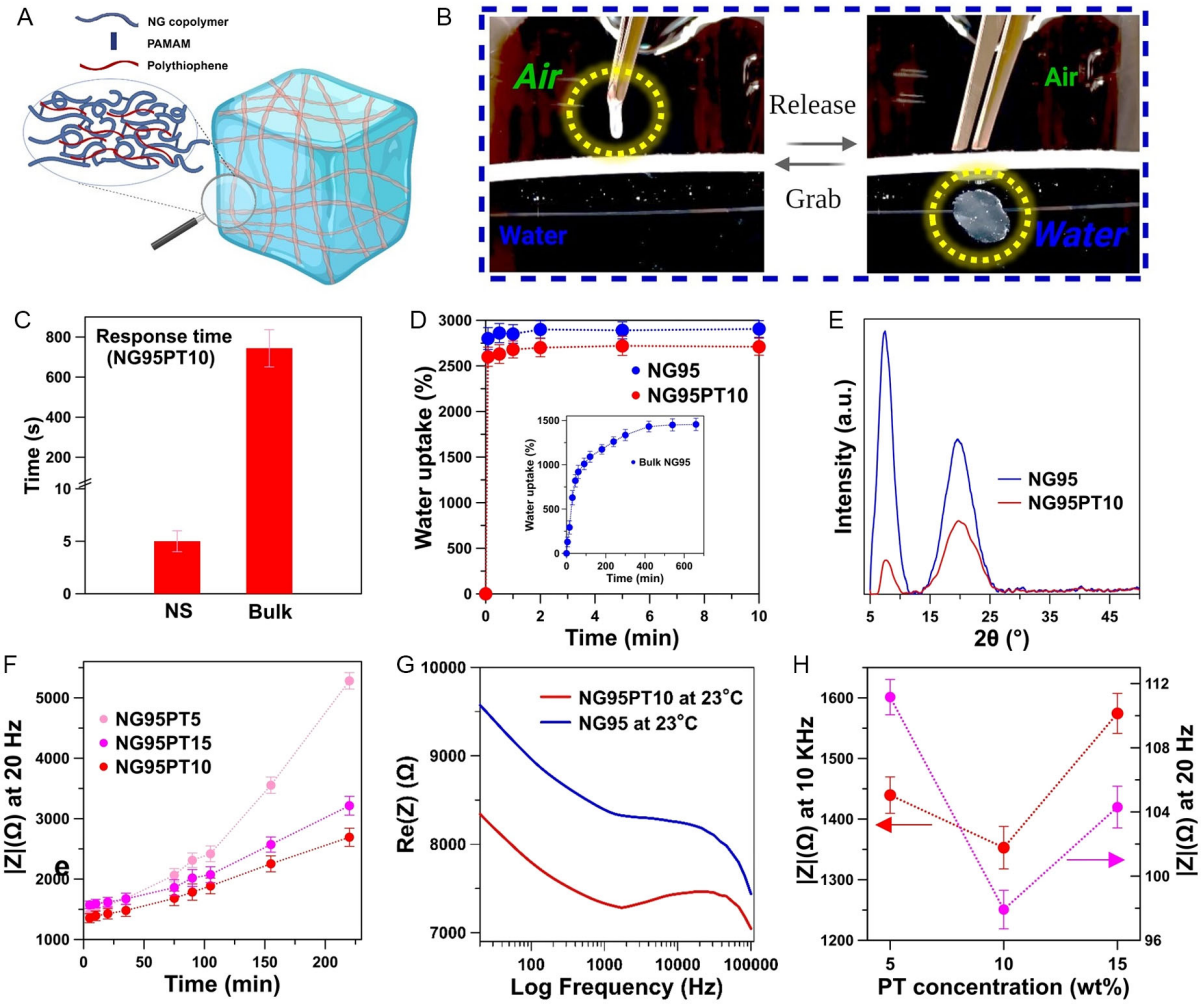
Structural and physical properties of the nanostructured hydrogels. A) Graphical representation of swelled NG95PT10 fibrous hydrogel showing a fast B) shape recovery and C) response time (NS: nanostructured). D) High water uptake of nanostructured hydrogels in comparison to the bulk form. E) XRD of fibrous hydrogels. F) Time evolution of impedance of semi‐IPN fibrous hydrogels. G) Representative graphs of the frequency‐dependent impedance of NG95 and NG95PT10 fibrous hydrogels at 23 °C. H) Dependence of the impedance on the PT6OMe concentration.

The transformation of the fibrous structure into a hydrogel state is schematically depicted in **Figure** **5**a. The fibrous hydrogels showed instant shape recovery (Figure 5b, Supporting Information video 1 and video 2). This remarkable phenomenon can be attributed to the unique structure of NG95 and NG95PT10 electrospun mats. The nanofibrous configuration offers several advantages over bulk hydrogels. One key advantage is the significantly enhanced water penetration rate into the hydrogel structure. Compared to bulk hydrogels, the nanofibrous architecture allows water molecules to infiltrate the material considerably faster (Figure 5c). It should be noted that the prevalence of moisture‐triggered shape memory behavior is gaining prominence in emerging applications of advanced biomaterials.^[^ ^35^, ^36^ ^]^ Furthermore, electrospun NG95 and NG95PT10 scaffolds exhibit a remarkable water‐absorbing capacity, outperforming their isotropic counterpart in terms of both the speed of hydration and the extent of swelling. Figure 5d shows that the fibrous NG95 has an exceptional water uptake, reaching a swelling ratio of ≈29 times its initial weight in less than 1 min, while the bulk hydrogel variant of NG95 significantly lags behind, achieving a maximum water uptake of ≈14 times its initial weight, and after a much longer period of 10 h. These observations demonstrate the benefit of nano‐structuration, which is manifested explicitly in the swelling of the NG95 fibrous hydrogel. It should be noted that the incorporation of PT6OMe within the NG95 hydrogel fibers resulted in a slight reduction of its water uptake capacity. PNIPAM hydrogels are well‐known for their chemical stability under physiological conditions. Specifically, those synthesized via free radical polymerization have been reported to exhibit poor biodegradability.^[^ ^37^, ^38^ ^]^ The slight hydrophobic characteristic of GMA comonomer and PT6OMe further reduces NG95PT10 susceptibility to biodegradation (Figure S9, Supporting Information). It also decreases the potential of PT6OMe to leach into aqueous environments, thereby preserving the electrical properties of the semi‐IPN NG95PT10 nanofibrous hydrogel.

We observed a higher relative intensity of the peak at 2*θ* ≈ 20° compared to the peak at 2*θ* ≈ 8.5° for the NG95PT10 XRD pattern (Figure 5e). This observation suggests that upon the introduction of PT6OMe to the NG chains, the crystalline portion of the semi‐IPN related to the PNIPAM‐based structure decreases. When two polymers lack compatibility, they tend to form separate crystalline regions within the blended films. As a result, X‐ray scans of these samples would essentially represent overlapping scans of the two polymers, preserving the initial blending ratio.^[^ ^39^ ^]^ However, in our case, the observation was indicative of the remarkable compatibility and intramolecular interactions between NG95 and the thiophenic polymer. Such miscibility plays a crucial role in shaping the material properties, specifically its electrical properties. Different percentages of polythiophene derivatives were mixed with NG95 copolymer (5, 10, and 15% by weight), and the resulting solutions were electrospun and cross‐linked in the presence of PAMAM.

As the initial step, changes in the impedance during the syneresis of semi‐IPN hydrogels were investigated (Figure 5f). As water escapes from the hydrogel network, ionic and electrical conductivity suffers. As a result, the impedance of all the hydrogels increased. Among the semi‐IPNs studied, the one denoted as NG95PT10 showed the lowest level of impedance. Interestingly, this sample clues to an optimum value of PT6OMe. Surpassing a certain threshold (in this case, 10 wt%) might induce partial macroscopic phase separation of thiophenic polymer from NG95. This, in turn, could have an adverse impact on the electrical properties of the semi‐IPN. The impedance of NG95PT10 was also compared to that of NG95 fibrous hydrogel (Figure 5g). As expected, the semi‐IPN hydrogel of NG95PT10 shows a significantly lower impedance when compared to bare PNIPAM‐based hydrogel. Figure 5h reveals how the amount of PT6OMe affects impedance at two different frequencies. It is important to note that all semi‐IPN hydrogels based on NG95 displayed impedances suitable for biomedical applications. Interestingly, NG95PT10 stood out by exhibiting the lowest impedance across the full range of frequencies under study. The electrochemical stability of NG95PT10 was also evaluated by conducting impedance measurements for a period of 10 days (Figure S10, Supporting Information). The results revealed a stable impedance profile at 20 Hz.

**Figure 6:**
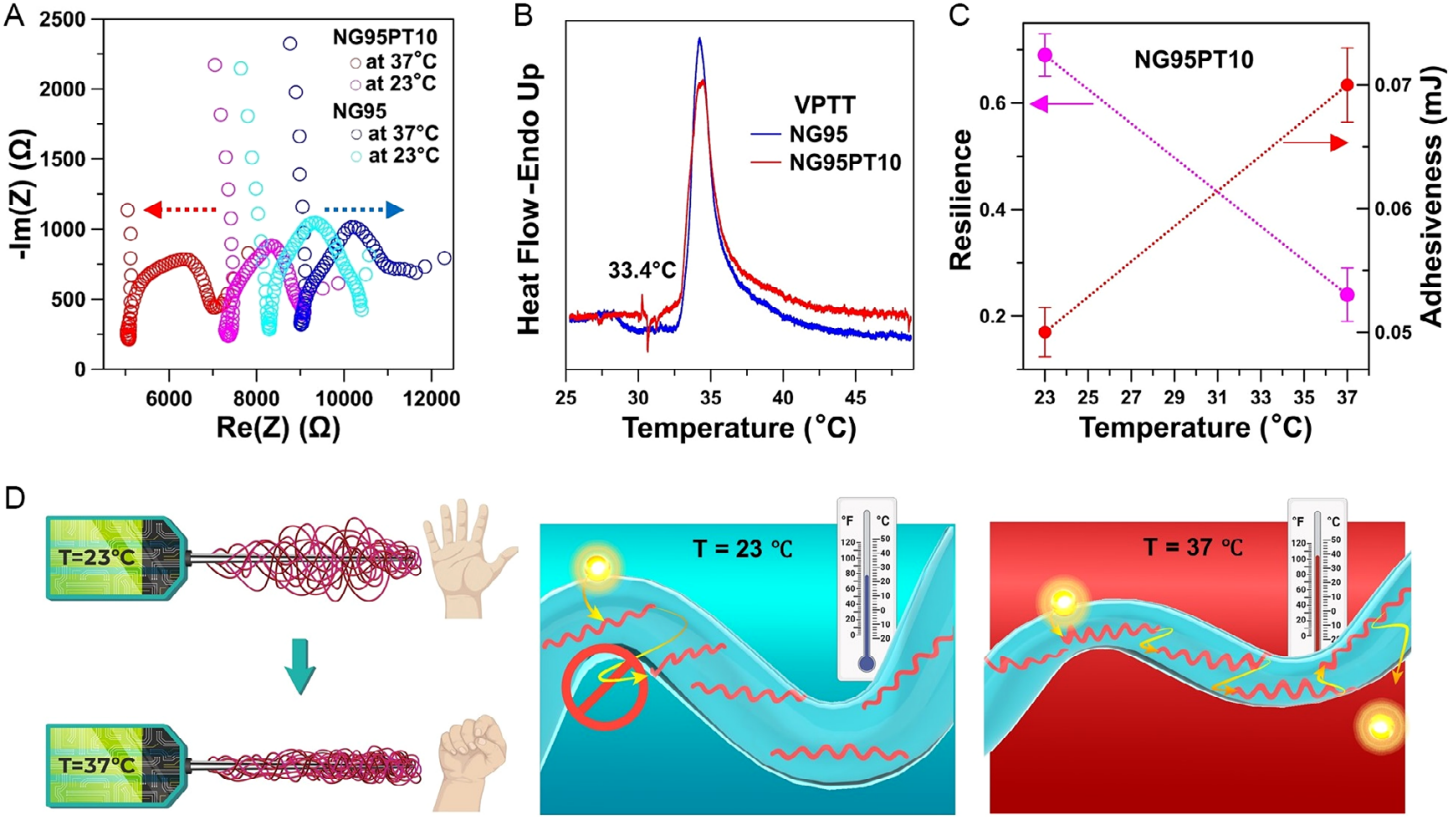
Functional properties of the nanoarchitectured fibrous hydrogels. A) Temperature dependence of NGPT95 and NG95 impedances: a reverse trend in favor of NGPT95. B) The active range of VPT temperature. C) Temperature dependence of NG95PT10 mechanical properties: a sacrifice in resilience for a better grip. D) Nanofibrous NG95PT10 hydrogel tightens its grip around the neural probe at body temperature while the packing of PT6OMe polymeric chains forms new conducting pathways.

To assess the electrical and mechanical properties of the semi‐IPN hydrogel at its intended working temperature, we conducted impedance evaluations and analyzed the resilience and adhesiveness of NG95PT10 and NG95 at different temperatures (**Figure** **6**).

PNIPAM hydrogels have been shown to exhibit a broad VPT behavior that spans over a wide temperature range, typically from 30 to 60 °C.^[^ ^40^ ^]^ Consequently, even though the hydrogel network within our semi‐IPN experiences some contraction at body temperature, it retains a sufficient degree of hydrophilicity. The shrinking of NG95PT10 at 37 °C might lead to a reconfiguration of PT6OMe polymeric chains, causing them to align within the fibers. This alignment could potentially enhance the development of conductive pathways within the material. To put our theory to the test, we conducted impedance measurements on both NG95 and NG95PT10 at 21 and 37 °C (Figure 6a). Surprisingly, we observed an inverse relationship between temperature and impedance for the semi‐IPN and NG95 hydrogels. While the impedance of circular NG95 hydrogel samples (1 cm diameter, ≈100 μm thick) increased from 8.33 ± 1.24 kΩ at 23 °C to nearly 9.06 ± 1.56 kΩ at 37 °C, the NG95PT10 impedance exhibited a notable drop, decreasing from 7.21 ± 1.35 kΩ at room temperature (RT) to 5.05 ± 1.09 kΩ at human body temperature. This finding supports our hypothesis that NG95PT10 becomes more conductive at body temperature owing to its semiconducting behavior.

We observed that the VPT temperature of the fibrous NG95 and semi‐IPN is equal to the LCST of the NG95 polymeric aqueous solution (Figure 6b). Utilizing a texture analyzer, we conducted evaluations on hydrogel samples under varying temperature conditions (Figure 6c). The resilience of NG95PT10 exhibited a notable decline, dropping from 0.69 ± 0.12 at RT to 0.24 ± 0.05 at 37 °C. This decrease in hydrogel resilience is closely linked to the extent of its syneresis (Figure S11, Supporting Information). NG95 is a temperature‐responsive hydrogel, which implies that it expels water from its NIPAM‐based structure when exposed to higher temperatures like 37 °C. With less water retained in the hydrogel structure, NG95PT10's ability to deform reversibly diminishes, thus explaining the lower resilience observed at body temperature. Conversely, the adhesiveness of the material increased from 0.05 ± 0.006 mJ at RT to 0.07 ± 0.005 mJ at 37 °C. The heightened adhesiveness of NG95PT10 as a coating for neural probes yields a twofold advantage. First, it results in a narrower gap between the coating and the probe, and therefore, it enhances the friction between the structural components of the neural probe and the coating.

Moreover, while the increased friction enhances the overall stability of the coating, it also ensures that the coating remains securely in place during the retrieval of the neural probe from the brain. Second, reducing the gap between the coating and the neural probe enhances signal transmission efficiency through the coating and onto the probe. Consequently, it can be expected that NG95PT10 will show suitable durability and electrical properties during chronic utilization, as demonstrated in Figure 6d.

### 2.4 Biocompatibility of Nanoarchitectured Fibrous Hydrogels

**Figure 7:**
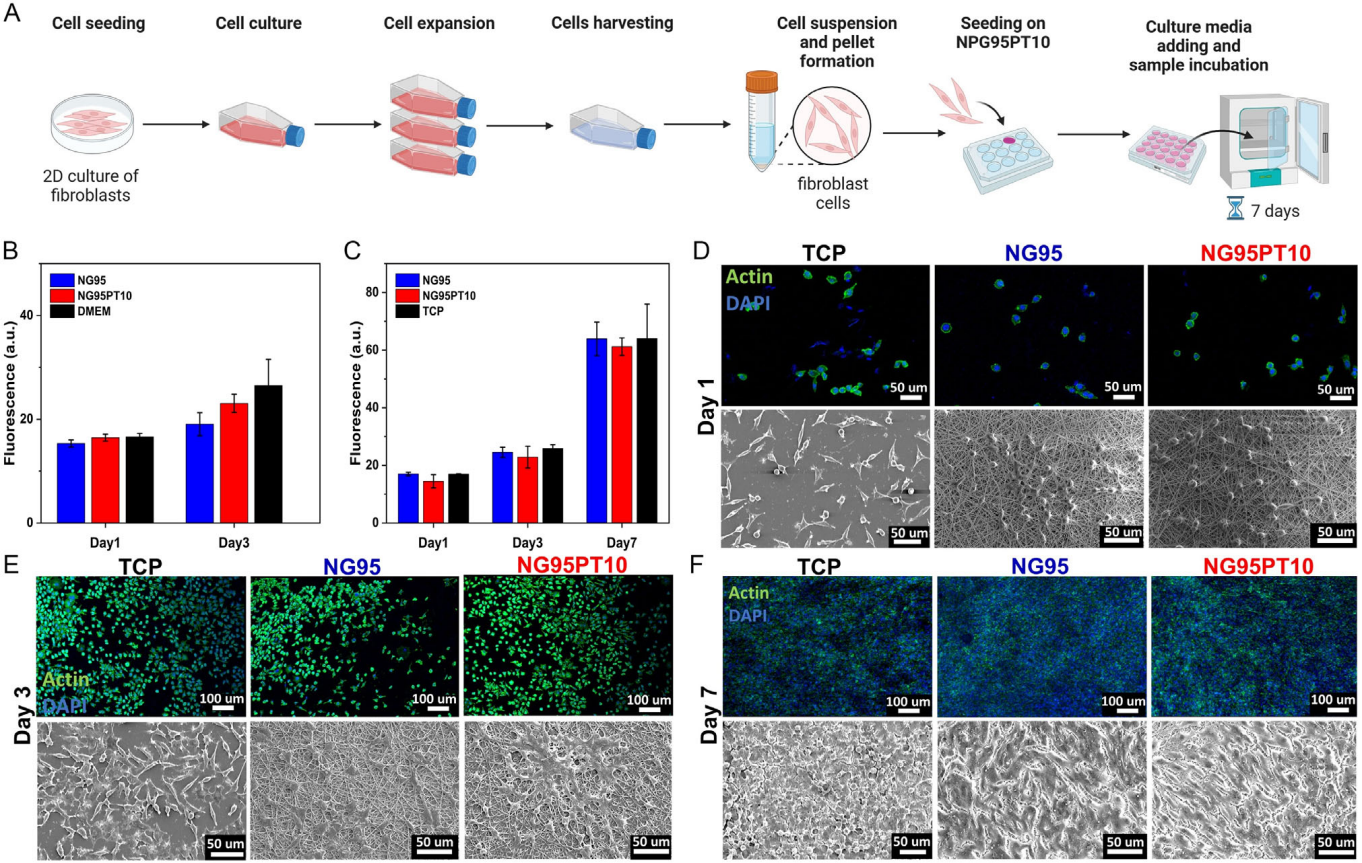
ISO standard guided nanofibrous hydrogel biocompatibility through L929 fibroblast cells viability. A) Schematic of cell culture and cell seeding. B) Indirect cytotoxicity of NG95 and NG95PT10 extracts and DMEM control up to 3 days of culture. C) Direct cytotoxicity of NG95, NG95PT10, and TCP control condition up to 7 days of culture. Morphology of L929 fibroblasts seeded on NG95, NG95PT10, and TCP control at day D) 1, E) 3, and F) 7 of culture. Confocal and SEM images were captured upon Actin/DAPI staining—to visualize cell cytoskeleton (green) and nuclei (blue)—or dehydration and gold sputtering, respectively.

To assess the biological properties of NG95 and NG95PT10 electrospun nanofibers, cytotoxicity tests were carried out using L929 fibroblasts following the ISO biocompatibility tests (**Figure** **7**a). In the case of the indirect test, the constructs were incubated in a culture medium for 24 h, and then extracts were added to L929 fibroblasts cultured on tissue culture plates. The effect of NG95 and NG95PT10 extracts were compared to cells cultured in Dulbecco's modified Eagle's medium (DMEM), revealing no significant differences in cell viability at both tested time points (Figure 7b). Additionally, cell growth was measured between days 1 and 3 of the culture. Thus, the direct cytotoxicity test was performed by seeding L929 fibroblasts on the materials. Results show a linear growth of the cells, which is demonstrated by increasing signal measurements at each time point (1, 3, and 7 days) for NG95, NG95PT10, and tissue culture plate (TCP) control (Figure 7c). Significant differences among the conditions were not reported at any time points, demonstrating the cytocompatibility of both materials. Cell morphology of fibroblasts seeded NG95 and NGPT10 was also analyzed and compared to TCP control on day 1 (Figure 7d), 3 (Figure 7e), and 7 (Figure 7f) of the culture. Actin/4′,6‐diamidino‐2‐phenylindole dihydrochloride (DAPI) staining and visualization under confocal microscope showed round cell cytoskeleton and nuclei at an early stage of the culture (day 1), followed by subsequent elongation and spreading of cells, which acquired the typical spindle shape at the initial stage (day 3). Finally, the proliferation and population of the whole electrospun matrix surface are observed (day 7). Results are supported by SEM images captured at the same time points (Figure 7d–f), where cell attachment, spreading, elongation, and proliferation can be observed on the NG95, NG95PT10, and TCP surfaces with no remarkable difference among the samples. Data showed the direct and indirect cytocompatibility of the NG95PT10 fibrous hydrogel, underlining its suitability for cell attachment, spreading, and proliferation and making it a safe candidate for implantation into the host body.

### 2.5 Design of the BMI Probe

**Figure 8:**
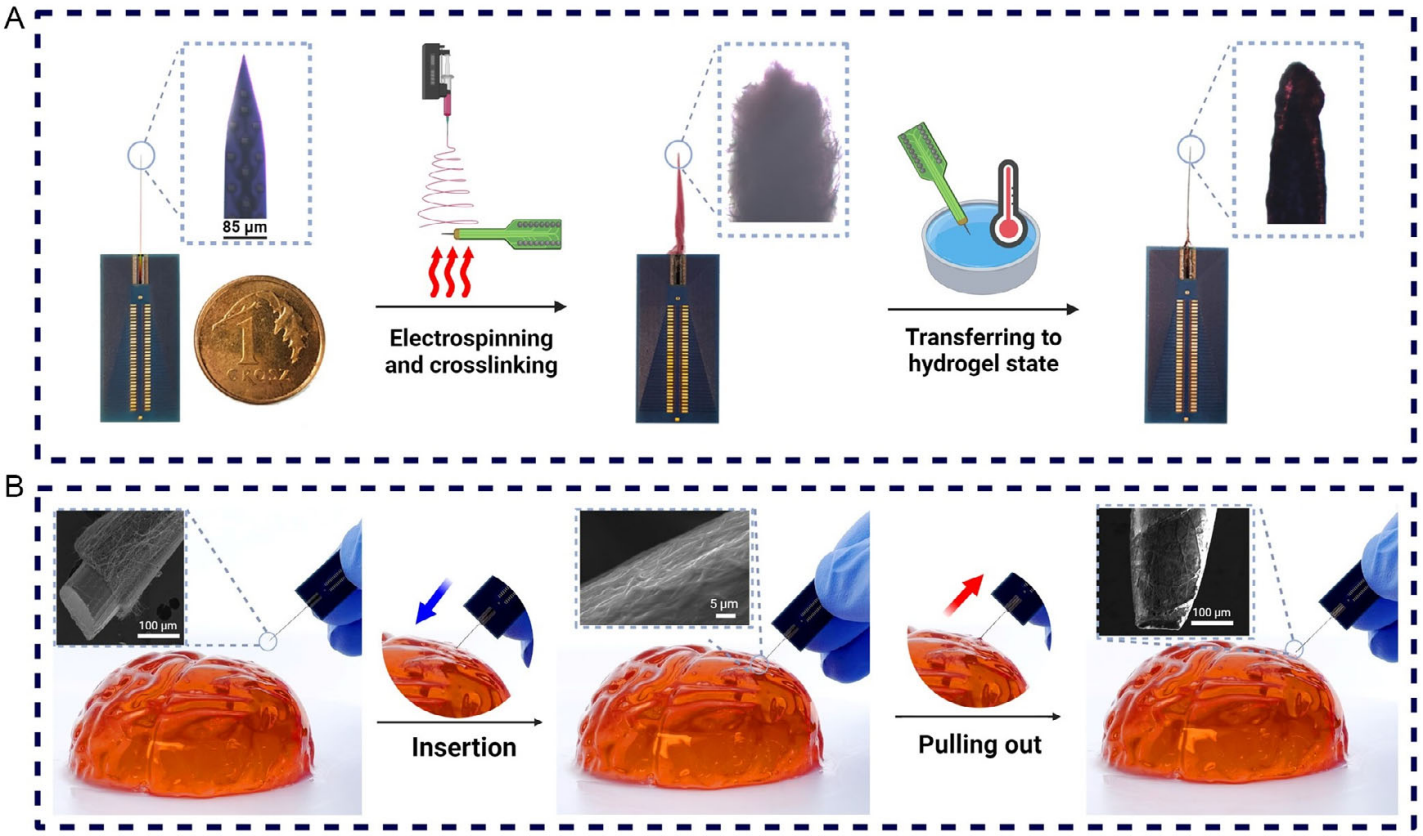
The design of the BMI probe and in vitro evaluation of the material insert ability using a hydrogel‐based brain phantom. A) Camera photo of the bare neural probe followed by coating the neural probe with NG95PT10 fibers and post‐cross‐linking. The coated tip of the neural probe was transferred into a hydrogel state before further demonstrations. B) The neural probe with a nanofibrous hydrogel coat of NG95PT10 was inserted in a gel with mechanical properties similar to the human brain. The SEM photo inserts show the integrity of the fibrous hydrogel coating after the neural probe is inserted and then pulled out from the gel.

After the evaluation of an optimal cytocompatibility level of our biomaterials, the design of the BMI probe via the combination of NG95, PT6OMe, and the microelectrode was commenced. A comprehensive demonstration of enveloping the surface of the neural probe with the electrospun NG95PT10 nanofibers and a subsequent cross‐linking process is shown in **Figure** **8**a. This experiment sought to match real‐world conditions where the coated BMI probe is introduced into the environment of brain tissue. To replicate this scenario, we employed a sodium alginate hydrogel, mimicking brain tissue's mechanical properties and consistency. As it transitioned from the electrospinning process to the insertion phase, our goal was to assess not only the adhesiveness and integrity of the semi‐IPN coating but also its ability to interface seamlessly with soft tissue. The BMI's probe, decorated with a robust NG95PT10 fibrous coating, was gently introduced into a brain‐shaped gel, representing the surrogate brain tissue environment. The insertion process was fully monitored, and as illustrated in Figure 8b, the semi‐IPN hydrogel section of the BMI's probe exhibited remarkable structural integrity throughout these steps. What adds to the success of this experiment is the subsequent retrieval of the BMI probe from the brain‐like gel. This process was executed without compromising the integrity of the fibrous hydrogel coating due to the nanostructured hydrogel's miniaturization and superior physical properties compared with the bulk materials. As depicted in the SEM photo inserts in Figure 8b, the fibers remained firmly adhered to the probe's surface, attesting to the effectiveness of our semi‐IPN as a coating material, which is not prone to be detached and left into the living tissue upon removal. This experiment serves as evidence of the practicality of our developed NG95PT10 semi‐IPN and its integration with neural probes during the applications within the complex parenchyma of brain tissue, setting an important innovation in terms of applicability.

### 2.6 Deep Brain Probing with the NG95PT10‐Coated BMI Probe

**Figure 9:**
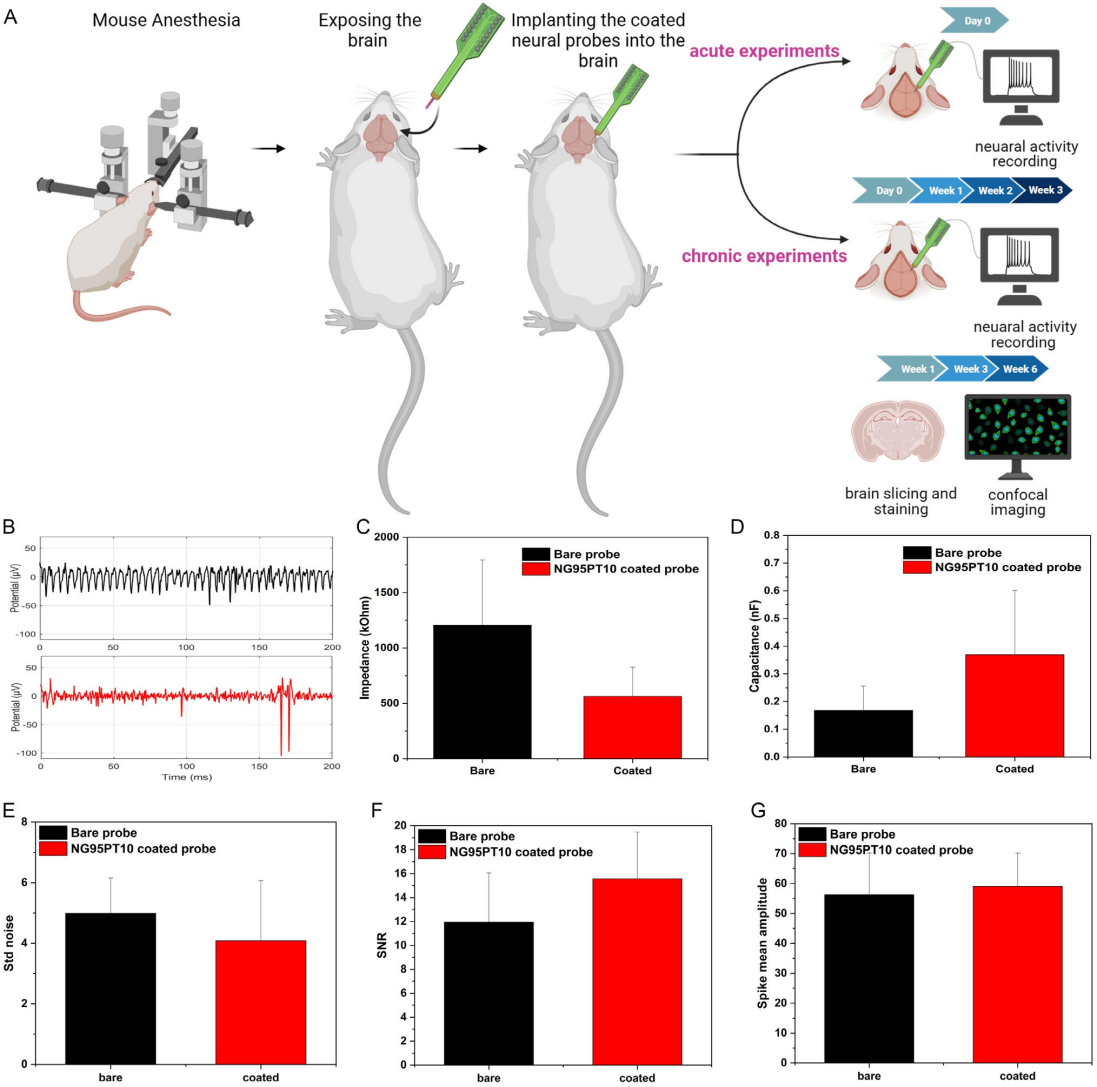
In vivo testing of the developed NG95PT10 semi‐IPN as a coating for neural probe applications. A) Schematic of in vivo experiments. Briefly, mice were anesthetized, and the brain tissue was exposed. Afterward, the NG95PT10‐coated neural probe and bare probe were inserted into the brain tissue, each in one hemisphere corresponding to the somatosensory thalamic regions. Recording acute experiments was carried out just after probe implantation, while chronic testing was performed at each selected time point for signal recording and biocompatibility evaluation. Acute recording through neural probes coated with NG95PT10 compared to bare probes: B) brain signal waveforms from the coated (red trace) and bare (black) platform showing good spikes detection on a low background noise in red but not black trace; C) impedance and D) capacitance values indicating the outperformance in terms of conductivity of the NG95PT10‐coated probes compared to bare ones; E) background noise (μV, i.e., standard deviation of a signal); F) SNR; and G) spike mean amplitude (μV) highlighting a trend for higher quality of the recorded signal through the design NG95PT10 fibrous structure.

Acute and chronic in vivo experiments were carried out to evaluate the performance of the neural probes coated with NG95PT10 within neuronal tissue in deep brain locations (**Figure** **9**a). Acute experiments were performed in terms of neuronal signal recording within the first hours post‐implantation, and consequently, key parameters were analyzed for NG95PT10‐coated neural probes in comparison with gold‐plated bare probes during early post‐implantation time. In addition to recording experiments, chronic tests also involved biocompatibility assessment and evaluation of neuroinflammation markers expression for up to 6 weeks post‐implantation.

Figure 9b shows the successful recording of the neuronal signal (clearly separated action potentials, also small ones) in the thalamus through the probe coated with the proposed material. Compared to bare neural probes, the NG95PT10 coating not only reports no adverse effect on the recorded signal but also shows a tendency of reduced impedance and improved capacitance (Figure 9c,d). Moreover, to support the claim of the beneficial effect of the coating with NG95PT10, it is worth mentioning that trends of lower background noise (visible also in Figure 9b) and higher signal‐to‐noise ratio (SNR) are observed (Figure 9e–g). All those technical parameters proved that the improvement given by applying the developed electrospun nanofibrous conductive hydrogel not only potentially impacts the bio integration of the BMI probe but also enhances the electrical interfacing between the system and the brain. Furthermore, post‐implantation observations confirm that NG95PT10 coating, after the retraction of coated neural probes from the brain, retains its fibrous architecture and structural integrity, adhering firmly to the probe (Figure S12, Supporting Information).

**Figure 10:**
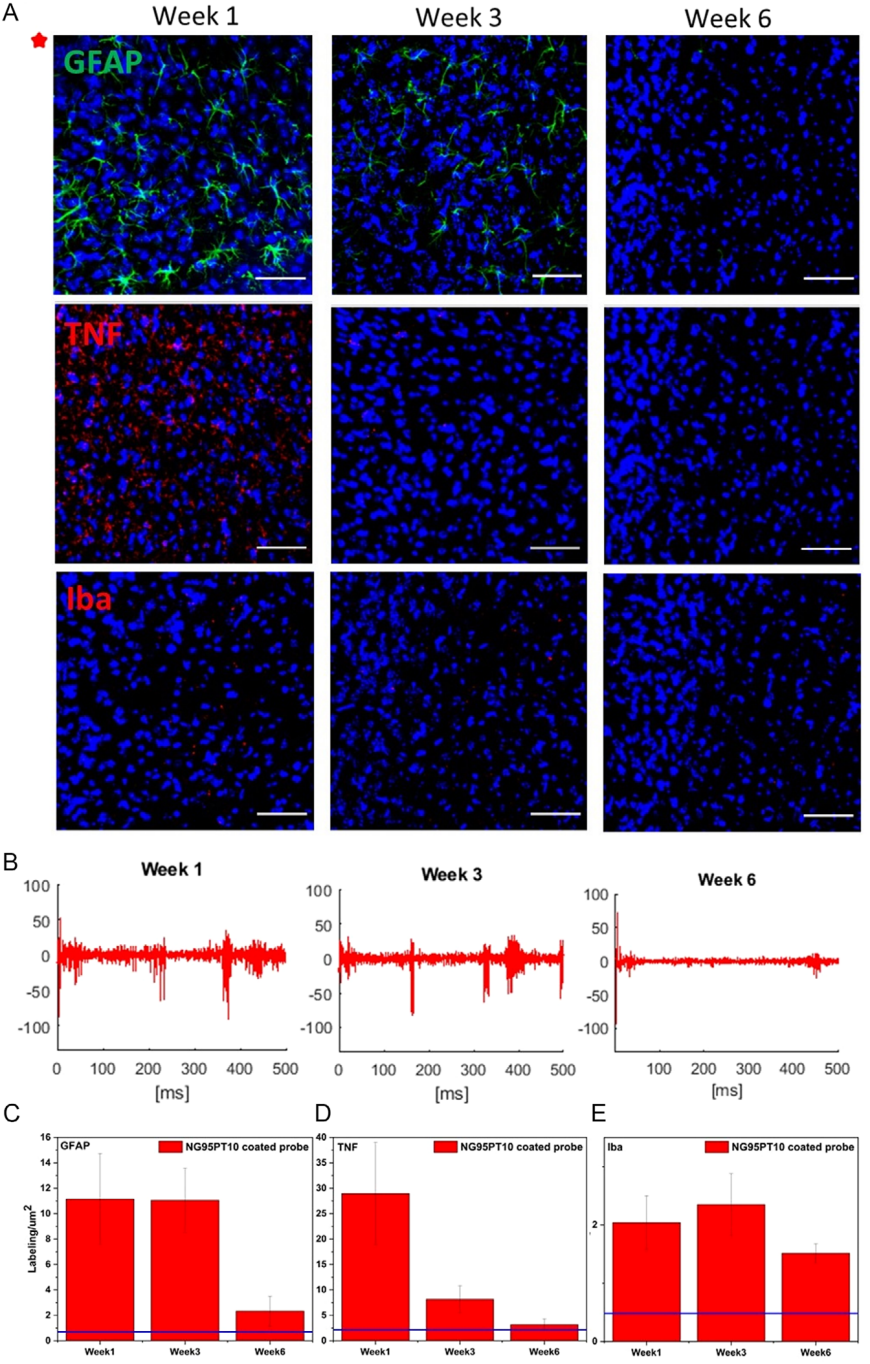
In vivo chronic experiments proving the developed NG95PT10‐based neural probe applicability. A) Confocal images of immunostained brain slices implanted for up to 6 weeks. Neuroinflammation markers GFAP (green color), TNF‐α (red color), and Iba‐1(red color) were immunolabeled. Cell nuclei were stained with DAPI (blue color). All images are oriented in the same manner. Scale bars: 40 μm. B) Chronic recording through bare neural probes: brain signal waveforms; Fluorescence quantification of C) GFAP, D) TNF‐α, and E) Iba‐1, plotted in comparison with untreated conditions (blue line).

Taking into account the promising acute results, chronic testing of NG95PT10‐coated neural probes and bare probes was performed at day 0 and 1, 2, 3, and 6 weeks after implantation (**Figure** **10** and S13–S15, Supporting Information). Our next goal was to estimate the effect of coating the silicon probe with NG95PT10 on the long‐term viability of the brain tissue affected by the surgical procedure of in vivo implantation. We analyzed the expression of three common markers of brain injury and inflammation for up to 6 weeks after in vivo implantation.^[^ ^41^, ^42^, ^43^ ^]^ We harvested the brains at 1, 3, and 6 weeks post‐surgery. We then immunolabeled glial fibrillary acidic protein (GFAP, a marker of astrogliosis), Iba‐1 (a marker of microglia activation), and tumor necrosis factor (TNF‐α; neuroinflammatory cytokine) in the brain tissue directly adjacent to the silicone electrode trace (Figure 10a). Comprehensive biocompatibility studies confirmed the lack of substantial chronic immune response to the newly synthesized copolymer coating, which was manifested by diminished reactive astrogliosis in the chronically implanted animals, as evidenced by gradually decreasing immunoreactivity against GFAP (Figure 10c). Also, the level of TNF‐α, an inflammatory cytokine secreted by monocytes and macrophages during chronic inflammation,^[^ ^41^, ^44^ ^]^ was reduced already at 3 weeks post‐surgery (Figure 10d). Iba‐1, another marker of macrophage activation,^[^ ^45^ ^]^ was only slightly elevated in the implanted animals, and the observed levels of this biomarker were substantially lower (an order of magnitude) than previously reported^[^ ^46^ ^]^ for injured mouse brains (Figure 10e). The results are consistent with the literature^[^ ^47^ ^]^ that shows a diminished brain reaction when the probe is inserted in the presence of a lubricating coating—despite the increase in diameter. Indeed, the lubricating coating served as a buffering medium, protecting the tissue from direct friction from a probe shaft.

The chronic recording through NG95PT10‐coated neural probes showed the efficient long‐term detection of neuronal activity in the thalamus (Figure 10b, S13, and S14, Supporting Information). The lack of recorded spikes at day 0 depends on the recovery anesthesia (using isoflurane is mandatory as the ethical commission required it) used during the implantation, which strongly dumped the neural activity. Instead, light anesthesia (i.e., ketamine with medetomide) used during later recording sessions (i.e., weeks 1, 2, 3, and 6) better preserved the neuronal activity. The decay in the spiking activity at week 6 is similar for coated and bare probes, consistent with previously reported data in the literature.^[^ ^48^ ^]^ The authors estimate that electrodes located posterior to the Bregma point in the rodent brain would show no spiking after 1 month of implantation due to neurons injured during implantation.^[^ ^48^ ^]^

In addition, the signal analysis indicates the stability of key parameters of the recording curves (i.e., impedance and capacitance), where the positive trend of both capacitance and impedance for coated probes versus bare probes is evident. More in detail, capacitance showed a trend of higher values in the case of NG95PT10‐coated neural probes compared to bare neural probes, while impedance indicated a trend of lower values for NG95PT10‐coated neural probes compared to bare neural probes at each tested time point (Figure S15, Supporting Information).

## 3 Conclusion

This work introduced a conducting semi‐IPN fibrous hydrogel‐based BMI probe designed to enhance the integration between microelectrodes and brain tissue during electrophysiological investigations. Our system uniquely integrates a cross‐linkable PNIPAM‐based copolymer with regioregular PT6OMe, fabricated via electrospinning and combined with a neural probe. The unique characteristics of the synthesized copolymer facilitated a seamless nanofabrication process, creating the fibrous structure and subsequent cross‐linking with a cytocompatible agent. The resulting fibrous semi‐IPN outperformed its isotropic counterpart in both the hydration speed and the swelling extent, demonstrating the benefits of nanostructuring. Furthermore, the smart semi‐IPN hydrogel demonstrated a unique response to body temperature. The partial shrinking of its network at 37 °C tightened the grip of the nanofibers around the neural probe. The mentioned increased adhesion and the optimal rearrangement of PT6OMe chains within the fibers resulted in improved electrical properties, as evidenced by decreased impedance at body temperature.

Acute in vivo tests showed favorable modulation of impedance and capacitance of our developed BMI probe for electrophysiological investigations. Critically, in vivo testing of NG95PT10‐coated probes demonstrated their ability to successfully detect deep brain signals, including neural spikes, in the somatosensory thalamic region—both in short and longer terms. This success highlights the platform's potential for chronic recording applications. Additionally, biocompatibility evaluation indicated a lack of significant inflammatory response, with a diminished reaction observed at later stages of implantation. These findings further validate the NG95PT10 semi‐IPN fibrous coating as a promising solution for implantable probes in chronic, high‐quality deep brain electrophysiological investigations. Thus, the proposed approach is an answer to the urging need for electrophysiological investigations, demonstrating the improvement of biocompatibility of the multielectrode probes for the better quality of recorded signal and improved protection of brain tissue. The proposed approach also highlights the system's potential for DBS, BMIs, and sEEG; thus, it holds promises to be applied for modulating the activity of diseased brain tissue (e.g., Parkinson's disease), signal recording from the motor cortex of paralyzed people, or epilepsy treatments, respectively.

Our research represents a significant advancement over previous studies by introducing a nanostructured hydrogel system that combines rapid swelling, fast shape recovery, and enhanced electrical performance at physiological temperatures, specifically tailored for coating BMI's rigid microelectrodes (Table S3, Supporting Information). It is important to emphasize that the mechanical properties of the developed hydrogel, in its standalone state, align with those of brain tissue, highlighting its suitability as a bio‐interface material. Overall, our work represented a significant step forward, addressing the limitations of conventional probes and opening new avenues for fully applicable, robust, reliable, and biocompatible BMI probes. The reduced mechanical mismatch achieved by the NG95PT10 coating not only enhances signal quality but also mitigates tissue inflammation, further strengthening the platform's potential for long‐term functionality.

## 4 Experimental Section

4.1

4.1.1

##### Materials

All reagents including *N*‐bromosuccinimide (NBS), 3‐bromothiophene, mequinol, 1,6‐dibromohexane, methylmagnesium bromide (a 3 M solution, in *n*‐butylether), dichloro(1,3‐bis(diphenylphosphino)propane)nickel (Ni(dppp)Cl_2_), (NIPAM (97%), APS, TEMED, PAMAM dendrimer (ethylenediamine core, generation 0.0, solution 20 wt% in methanol), GMA (≥97.0% [GC]), bovine serum albumin (BSA), hexamethyldisilazane (HMDS), phosphate buffer saline (PBS), glutaraldehyde (GTA), Triton X‐100, and DAPI were purchased from Aldrich Chemical Co. and used without further purification otherwise indicated. Solvents such as DMF, *n*‐hexane, diethyl ether, tetrahydrofuran (THF), *n*‐hexane, methanol, chloroform were purified by standard procedures, stored under molecular sieves, and handled in a moisture‐free atmosphere. L929 murine fibroblasts were obtained from Sigma‐Aldrich. DMEM, fetal bovine serum (FBS), penicillin streptomycin (PS), Fluromount with DAPI (#00‐4959‐52), and ethylenediaminetetraacetic acid–trypsin (EDTA–trypsin) were purchased from Gibco Invitrogen. Alexa Fluor 488 Phalloidin and PrestoBlue reagents, rabbit polyclonal anti‐GFAP (#PA5‐16291), rat monoclonal anti‐TNF‐α (#14‐7321‐85), mouse monoclonal anti‐Iba‐1 (#MA5‐27726), Alexa Fluor 488 goat anti‐rabbit (#A48282), Alexa Fluor 647 goat anti‐rat (#A48265), Alexa Fluor 568 goat anti‐mouse (#A‐11031) were obtained from Thermo‐Fisher Scientific. Normal goat serum (NGS, #S‐1000) was obtained from Vector Laboratories.

##### Synthesis, Material Fabrication, and Characterization: Synthesis of BT6OMe

An amount of 2.02 g (11.3 mmol) of NBS into 11 mL of anhydrous DMF were added dropwise to a solution of 2.25 g (11.3 mmol) of 3‐(6‐methoxyhexyl)thiophene (T6OMe) prepared as reported in ref. [49] in 11 mL of DMF under argon atmosphere. The reaction mixture was stirred for 6 h at 20 °C in the dark under an inert atmosphere. A second amount of NBS (3.02 g in 17 mL of DMF) was then added to the system and stirred for a further 24 h at 20 °C in the dark and under an inert atmosphere. The mixture was poured into 300 mL of distilled water, extracted with *n*‐hexane (3 × 100 mL), dried, and evaporated under reduced pressure. The crude oil obtained was purified by chromatography on silica gel (n‐hexane/diethyl ether 4:1) to give pure BT6OMe as a colorless oil (2.95 g, 8.28 mmol, 73% yield).

^1^H‐NMR (400.13 MHz, CDCl_3_,): *δ* 6.78 (s, 1H, Th H‐4), 3.35 (m, 5H, CH_2_OCH_3_), 2.51 (t, 2H, ThCH_2_), 1.55 (m, 4H, ThCH_2_CH_2_ and CH_2_CH_2_OCH_3_), 1.32 (m, 4H, (CH_2_)_2_) ppm.

^13^C‐NMR (100.61 MHz, CDCl_3_): *δ* 143.52 (Th C‐3), 131.61 (Th C‐4), 111.02 (Th C‐2), 108.65 (Th C‐5), 73.45 (CH_2_OCH_3_), 59.22 (CH_2_OCH_3_), 30.19 (ThCH_2_CH_2_), 30.16 (ThCH_2_CH_2_CH_2_), 30.04 (ThCH_2_CH_2_CH_2_CH_2_), 29.57 (ThCH_2_), 26.55 (CH_2_CH_2_OCH_3_) ppm.

FT‐IR (KBr, cm^−1^): 3043 (ν_C—H_ Th), 2978 (ν_as_ —CH_3_), 2932 (ν_as_ —CH_2_—), 2856 (ν_s_ —CH_2_—), 1542 (ν_as_ Th C=C), 1460 (ν_s_ Th C=C), 1391 (—CH_3_ def.), 1120 (ν C—O—C), 1000 (ν aromatic C—Br), 824 (γ —CH Th), 726 (rocking —CH_2_—).

##### Synthesis, Material Fabrication, and Characterization: Synthesis of Regioregular PT6OMe

In a three‐neck round bottom flask equipped with a perforable septum, a 3 M solution of methylmagnesium bromide in *n*‐butylether (2.67 mL, 8.02 mmol) was added using a syringe to a solution of 2.84 g (7.97 mmol) of BT6OMe in 50 mL of anhydrous THF. The mixture was stirred at reflux for 1 h under a gentle flux of argon, and then 0.046 g (0.085 mmol) of Ni(dppp)Cl_2_ was added. After refluxing for a further 2 h under an inert atmosphere, the mixture was cooled down at RT and poured into 400 mL of methanol. The obtained dark‐purple precipitate was filtered on a polytetrafluoroethylene septum (0.20 μm pore size), and the recovered polymer was redissolved in 20 mL of chloroform, precipitated in 200 mL of methanol and filtered on a glass septum to give 1.21 g (6.16 mmol, 77% yield) of PT6OMe.

^1^H‐NMR (400.13 MHz, CDCl_3_): *δ* 6.97 (s, 1H, Th H‐4), 3.38 (t, 2H, CH_2_OCH_3_), 3.33 (s, 3H, CH_2_OCH_3_), 2.81 (m, 2H, ThCH_2_), 1.72 (m, 2H, ThCH_2_CH_2_), 1.61 (m, 2H, CH_2_CH_2_OCH_3_), 1.44 (m, 4H, (CH_2_)_2_) ppm.

^13^C‐NMR (100.61 MHz, CDCl_3_): *δ* 139.93 (Th C‐3), 133.84 (Th C‐4), 130.69 (Th C‐2), 128.79 (Th C‐5), 73.00 (CH_2_OCH_3_), 58.72 (CH_2_OCH_3_), 30.67, 29.77, 29.58 (ThCH_2_CH_2_CH_2_CH_2_), 29.52 (ThCH_2_), 26.19 (CH_2_CH_2_OCH_3_) ppm.

FT‐IR (KBr, cm^−1^): 3053 (ν_C—H_ Th), 2975 (ν_as_ —CH_3_), 2930 (ν_as_ —CH_2_—), 2855 (ν_s_ —CH_2_—), 1512 (ν_as_ Th C=C), 1478 (ν_s_ Th C=C), 1388 (—CH_3_ def.), 1121 (ν C—*O*—C), 822 (γ —CH Th), 727 (rocking —CH_2_—).

Elemental analysis (C_11_H_16_OS): calculated: C 67.30, H 8.22, O 8.15, S 16.33; found: C 66.93, H 8.12, O 8.49, S 16.46.

##### Synthesis, Material Fabrication, and Characterization: Copolymerization of NG

Copolymers of NIPAM and GMA were synthesized through a free radical polymerization process, beginning with the dissolution of the monomers in deionized water. Different weight ratios of these monomers were carefully chosen, and the resulting copolymers were named based on the initial NIPAM content in the feed, yielding NG70, NG90, NG93, NG95, and NG97 when the NIPAM weight percentages were 70, 90, 93, 95, and 97%, respectively. The copolymerization reaction was conducted in a glass reactor equipped with a perforable septum. APS was employed after eliminating oxygen by purging the reactor with nitrogen gas to initiate the reaction. Additionally, a redox initiator system was established by introducing TEMED, a tertiary diamine, into the mixture. During the copolymerization process, the reaction mixture exhibited distinct behaviors. For instance, NG97 maintained its transparency throughout, while NG90 and NG70 formed gummy‐like agglomerates. In the cases of NG95 and NG93, the mixture initially became turbid but gradually regained transparency after 10 and 25 min, respectively. After ≈4 h, the resulting viscous solutions were placed in dialysis bags and left for 10 days to eliminate any unreacted substances. Subsequently, the dialyzed solutions were lyophilized. To prevent undesired reactions between the functional groups of NG, the resulting white, foamy copolymers were stored in a dry environment at 4 °C for future use.

**NG97** ^1^H‐NMR (400 MHz, dimethyl sulfoxide (DMSO)‐d_6_, ppm): *δ* 7.18 (bm, 1H, l‐H), 4.78 and 4.27 (bm, 2H, i‐H), 3.84 (bm, 1H, h‐H), 3.23 (bm, 1H, g‐H), 2.81 and 2.66 (bm, 2H, f‐H), 1.96 (bm, 3H, d‐H and e‐H), 1.43 (bm, 2H, c‐H), 1.23 (bs, 3H, b‐H), and 1.04 (bm, 6H, a‐H).

**NG95** ^1^H‐NMR (400 MHz, DMSO‐d_6_, ppm): *δ* 7.19 (bm, 1H, l‐H), 4.79 and 4.25 (bm, 2H, i‐H), 3.84 (bm, 1H, h‐H), 3.23 (bm, 1H, g‐H), 2.80 and 2.67 (bm, 2H, f‐H), 1.96 (bm, 3H, d‐H and e‐H), 1.44 (bm, 2H, c‐H), 1.23 (bs, 3H, b‐H), and 1.04 (bm, 6H, a‐H).

**NG93** ^1^H‐NMR (400 MHz, DMSO‐d_6_, ppm): *δ* 7.19 (bm, 1H, l‐H), 4.78 and 4.27 (bm, 2H, i‐H), 3.84 (bm, 1H, h‐H), 3.23 (bm, 1H, g‐H), 2.80 and 2.66 (bm, 2H, f‐H), 1.96 (bm, 3H, d‐H and e‐H), 1.43 (bm, 2H, c‐H), 1.23 (bs, 3H, b‐H), and 1.04 (bm, 6H, a‐H).

##### Synthesis, Material Fabrication, and Characterization: Bulk and Fibrous Hydrogel Fabrication

NG95 copolymer and PAMAM dendrimer were combined in specific quantities and dissolved in either water or chloroform to fabricate bulk hydrogels. The NG95 to PAMAM weight ratio was maintained at 80% w/w, while the NG95 to PT6OMe weight ratio was set at 90% w/w. After solvent removal, the samples were subjected to a 2 h cross‐linking process at 53 °C in an oven. The samples were then allowed to cool down to RT, and water was added to the cross‐linked materials to induce their transformation into hydrogels. The resulting hydrogels were designated as NG95‐bulk hydrogel and NG95PT10‐bulk hydrogel to indicate the presence or absence of PT6OMe explicitly.

To create the fibrous hydrogels, NG95 copolymer was blended with PAMAM dendrimer and supplemented with PT6OMe in various weight ratios (5, 10, and 15% w/w). A chloroform/DMF solvent system (75/25 v/v) was employed to maintain a balance between the volatility and conductivity of the electrospinning solution, with the final concentration of NG95 maintained at 7% w/v. These solutions were loaded into a syringe and subjected to a high voltage of 13 kV. Employing a flow rate of 750 μL h^−1^, a tip to distance of 13 cm, and a 20G needle tip, the electrospinning process was carried out at RT with 50% humidity. The electrospun samples were then transferred to an oven and heated at 53 °C for 2 h to cross‐link the fibrous constructs. The samples were then removed from the oven and allowed to cool down to RT before the addition of water. The resulting fibrous hydrogels were designated as NG95PT5, NG95PT10, and NG95PT15, indicating the corresponding weight ratio of PT6OMe. The same electrospinning and cross‐linking procedure was also carried out for the neat solution of NG95 in a chloroform/DMF solvent system (75/25 v/v) with a concentration of 7% w/v to yield NG95 fibrous hydrogel.

##### Synthesis, Material Fabrication, and Characterization: Spectroscopic and Morphological Characterization

^1^H‐NMR and ^13^C‐NMR spectra were recorded with a Varian Mercury 400 (400 MHz) spectrometer; chemical shifts are given in ppm and were determined relative to the ^1^H and ^13^C resonance shift of solvents used: CDCl_3_ (^1^H: 7.26 ppm, ^13^C: 77.00 ppm) and DMSO‐d_6_ (^1^H: 2.50 ppm).

UV–Vis analyses were carried out on PT6OMe polymer solution in spectroquality chloroform at a concentration of 5 × 10^−5^ mol L^−1^ and on a polymer film cast on a quartz slide using a Perkin Elmer Lambda 19 spectrophotometer. A film was prepared by casting 0.25 mL of a 5 × 10^−4^ M solution based on the examined polymer in chloroform on a 3 × 1 cm quartz slide.

T6OMe and PT6OMe FT‐IR spectra were recorded using KBr‐based pellets on a Perkin Elmer Spectrum One spectrophotometer. NG bulk copolymers, electrospun NG95, and NG95PT FT‐IR analyses were carried out in attenuated total reflectance mode with a Bruker Vertex70 FT‐IR spectrometer and wavenumber range of 2000–800 cm^−1^ was recorded with a resolution of 2 cm^−1^ after 12 scans for each sample.

Molecular weight and polydispersity were determined by GPC analysis, with a polystyrene standards calibration, by using THF solutions on an high‐performance liquid chromatography Lab Flow 2000 apparatus equipped with a Rheodyne 7725i injector, a Phenomenex Phenogel MXM 5 μm mixed bed column, and an RI K‐2301 KNAUER detector.

A DSC TA Instruments 2920 was used for the thermal analysis of PT6OMe. Temperatures ranged from –50 to 250 °C with a rate of 10 °C min^−1^. The reported thermogram refers to the second cycle under the nitrogen atmosphere. The Pyris 1 DSC PerkinElmer calorimeter (USA, Waltham) was used to perform DSC measurements of NG95 aqueous solution as well as NG95 and NG95PT10 fibrous hydrogels. The measurements were carried out under non‐isothermal conditions, in the 20–70 °C temperature range, with a constant heating–cooling rate of 0.5 °C min^−1^. Each sample was placed in a dedicated hermetic pan to prevent water evaporation. The measurements were conducted with a reference water sample of similar mass and were run for three cycles, with the results averaged.

A TGA using a TA Instruments 2050, operated in an oxidizing (air) atmosphere, was employed to determine the decomposition temperatures of PT6OMe. Thermograms were recorded by heating samples from 40 to 900 °C with a heating scan rate of 20 °C min^−1^. In the case of NG95 and NG95PT10, a TG 209 F3 Tarsus apparatus (Netzsch) was used for the TGA. The heating rate was 10 °C min^−1^ under a nitrogen atmosphere, in the temperature range from RT to 800 °C.

All samples were imaged using an SEM (JSM‐6010PLUS/LV, In TouchScope microscope). Bulk and fibrous hydrogel samples were freeze‐dried. Before imaging, all samples were sputtered with gold layers of ≈8 nm in thickness using a SC7620 Polaron mini sputter coater (Quorum Technologies Ltd., Ashford, UK). Images were taken with different electron beam energies (7–12 kV) and magnifications, depending on the sample.

XRD data of PT6OMe films were recorded at RT by using a CuKα (*λ* = 1.5406 Å) radiation source (Philips PW 1050) and a Bragg–Brentano diffractometer (Philips PW 1710) equipped with a graphite monochromator in the diffracted beam. The 2*θ* range between 2.0° and 90.0° was scanned by 881 steps of 0.1° with a counting time of 15 s for each step. Using Bragg–Brentano geometry, XRD measurements of NG copolymers and fibrous samples were performed on a Bruker D8 Discover diffractometer. The analysis was conducted in the angular range of 5°–50° (2*θ*), with data being collected at each point using a step size of 0.02° per 1.0 s.

##### Synthesis, Material Fabrication, and Characterization: Electrical Properties

The resistivity of the PT6OMe polymer film was measured using an Alessi Instruments C4S four‐point probe head (probe material: osmium; probe tip spacing: 1.6 mm; spring pressure: 40–70 g; probe tip diameter: 0.2 mm) connected to a source meter (Keithley 2401). The polymer was dissolved in chloroform (10 mg mL^−1^), and the obtained solution was drop‐casted on a glass substrate. The film was then dried, and its measured thickness was about 10 μm. The measurement was performed in a vacuum (10^−3^ mmHg) and in an ambient atmosphere. Impedance measurements were conducted using a Keysight E4990A impedance analyzer (Keysight Technologies). For each sample, three circular specimens were chosen from the electrospun mats of NG95, NG95PT5, NG95PT10, and NG95PT15, each having a 1 cm diameter and an average thickness of ≈100 μm. These specimens were transformed into their hydrogel state and carefully positioned between two conductive indium tin oxide (ITO) glass slides (coated with ITO, Sigma, with a resistance of 15−25 Ω sq^−1^), which were subsequently connected to the impedance analyzer. The frequency range employed for impedance measurement ranged from 1 Hz to 100 kHz. Galvano electrochemical impedance spectroscopy (EIS) A potentiostat (SP‐50e, BioLogic) equipped with a probe station with a four‐point probe (KSR‐4, Everbeing Int'l Corp.) was used for EIS of NG95 and NG95PT10 fibrous hydrogels. Samples were tested while being in a hydrogel state and at different temperatures. The control range was between −10 to +10 V. A 0.5 mA current was selected, and three cycles were repeated for each sample.

##### Synthesis, Material Fabrication, and Characterization: Hydrogel–Water Relationship Evaluation

Water absorption, retention, and response time were evaluated for both bulk and fibrous cross‐linked samples to assess their water‐absorbing capacity and retention abilities. Initially, the dry weight of the samples (W0) was determined. Subsequently, the samples were immersed in water and weighed at various time intervals (1–20 min for fibrous samples) (W1). The water absorption was quantified as a percentage, calculated by subtracting W1 from W0 and then dividing by W0. Moreover, the time required for bulk and fibrous samples to reach their equilibrium hydrogel state was measured, identifying the point at which maximum water absorption occurred. Following this, the hydrogels at their equilibrium state were allowed to dry in ambient air at RT, and the sample weights were recorded once again.

##### Synthesis, Material Fabrication, and Characterization: Mechanical Properties

The dynamic thermomechanical analysis (DMA) was performed in shear mode on a DMA1 Mettler Toledo instrument. The hydrogel materials were measured in the form of cylindrical samples measuring 10 mm in diameter and 0.4 mm in thickness in a standard sample holder for shearing mode. To investigate the viscoelastic properties of the hydrogels, a frequency sweep experiment was performed. The isothermal measurements were conducted at the frequency range from 0.1 to 1000 Hz with 10 points per decade, at constant temperatures (*T* = 25 °C) and 5 μm displacement amplitude (shear strain 1%). To calculate the resilience and adhesiveness of the samples, a CTX Texture Analyzer, Brookfield Ametek, was used. The device was equipped with Texture Pro V1.0 Build 19 software. Samples were cut into circular shapes (2 cm in diameter) and were put in a water bath fixed in the lower jaw of the device. A circular silicon wafer with the same size was fixed to the upper jaw. These two specimens were allowed to touch each other at the force of 1 N for 60 s at RT and 37 °C. The force needed to detach the silicon wafer from the hydrogel samples was measured, and the resilience and adhesiveness were derived from the measurement.

##### Cell Studies: L929 Fibroblast Culture and Sterilization of the Samples

Murine L929 fibroblast cells were cultured in the presence of DMEM modified with 10% FBS and 1% PS and incubated at 37 °C and 5% CO_2_. The culture medium was changed every other day. When the cell confluence reached ≈80%, cells were detached and passaged by adding 0.05% EDTA–trypsin. Cells were then incubated for 3 min at 37 °C and 5% CO_2_. Afterward, cells were collected and centrifuged at 1200 rpm for 5 min to form a cell pellet. After discarding the supernatant, cells were resuspended in a 1 mL culture medium for counting. Lastly, the cell suspension was further diluted in culture media to obtain the desired cell density for sample seeding.

NG95 and NG95PT10 fibrous structures were electrospun onto coverslips (diameter = 1.5 cm) and cross‐linked. Then, each side of the samples was sterilized by UV light exposure for 30 min.

##### Cell Studies: Direct Cytotoxicity Test

Samples were located in 24‐well plates, and L929 fibroblasts were seeded on top of the electrospun materials at a density of 104 cells cm^−2^. A control condition was also assessed by seeding the same cell density on TCPs. All the sample conditions were incubated and cultured for up to 7 days.

##### Cell Studies: Indirect Cytotoxicity Test

Cells were seeded in 24‐tissue culture plates at a density of 10^4^ cells cm^−2^, incubated for 24 h in a culture medium to allow an optimal attachment to the well bottom, and used the following day for the test. In the meantime, fibrous samples were then incubated for 24 h in a calculated volume of culture medium according to their area as suggested by the ISO standard 10993‐12 (6 cm^2^ mL^−1^). The following day, extracts were collected and filtered through a 0.22 μm filter and used to replace cell culture media in contact with the cells seeded on the tissue culture plate. A control condition was also assessed by using fresh DMEM.

##### Cell Studies: Cell Viability

The PrestoBlue assay was applied to evaluate the viability of cells after 1, 3, and 7 days of contact with the proposed materials. The reagent was prepared in a concentration of 10% v/v in culture media and added on NG95 and NG95PT10 fibrous materials, and TCP seeded with fibroblasts. Five replicates of each sample were incubated for 1 h at 37 °C and 5% CO_2_. After the incubation time, PrestoBlue aliquots of 100 μL were transferred to a 96‐well plate and measured using a fluorometer plate reader at excitation 530 nm and emission at 620 nm (Fluoroskan Ascent TM Microplate Fluorometer, Thermo Scientific).

##### Cell Studies: Cell Morphology

L929 fibroblast morphology in contact with NG95, NG95PT10, and TCP was investigated by actin/DAPI staining and imaging. The cytoskeleton and nuclei of the sample were stained in triplicates for 1, 3, and 7 days of cell culture. The protocol provides the fixation of samples in 4% paraformaldehyde for 15 min at RT, followed by three washing steps in PBS. Afterward, 0.3% v/v of Triton X‐100 was added for 15 min, and washing steps were repeated. Then, samples were treated with a solution of 1% w/v BSA in PBS for 30 min. Alexa Fluor 488 Phalloidin solution (1:40) was then added, and samples were incubated at RT for 40 min in the dark. Finally, nuclei staining was assessed by adding a solution of 1:500 DAPI for 10 min. Three washing steps in PBS were carried out, and the constructs were imaged using a confocal microscope (Leica).

Additional evaluation of cell morphology was performed using SEM. Samples were analyzed in triplicates after 1, 3, and 7 days of culture by fixing in 3% ice‐cold GTA for 3 h. Samples were then washed in DI water three times and then dehydrated by adding increasing ethanol concentrated solution (15 min each): 50, 70, 90, and 100%. Afterward, the constructs were treated with HMDS and dried overnight under a fume hood. Finally, samples with a thin layer of gold were sputtered, and samples were imaged with SEM.

##### In Vivo Experiments: Silicon Probes

In vivo tests were performed using silicon probes from the Sotiris Masmanidis laboratory.^[^ ^50^ ^]^ For histological (immunocytological) evaluation of biocompatibility, only spare silicone shafts of the probes were used. Full probes (64Dxl design) were used for electrophysiological recordings with head‐stage connectors (see Figure 8a). The probes for acute experiments required isolation of wire bonding with Resinlab EP965 Black epoxy (as instructed in the probe designer guide—microprofe_info.pdf from https://masmanidislab.neurobio.ucla.edu/technology.html). The same probe design was used for the longitudinal experiment but in a commercially offered by White Matter LLC “chronic” assembly, with a double Molex connector on a 3 cm long flexible ribbon cable. Contacts were gold electroplated (non‐cyanide gold solution, Sifco) to reduce their impedance and noise level. Half of the probes have been coated with gel fibers along their entire length, including the 1 mm tip on which recording points are located. Electrospinning enabled precise control over the coating thickness (Figure S16, Supporting Information).

##### In Vivo Experiments: Animals and Surgery

A total of 18 adult (3–6 months old) male C57BL/6 mice were used for in vivo histological and electrophysiological tests. Mice were obtained from and housed in the Nencki Institute animal house in a RT with controlled temperature, humidity, ventilation, and non‐reversed daylight (lights off between 8:00 PM and 8:00 AM). Mice were kept in cages with 2–3 littermates before surgery and housed separately in the same environment post‐surgery. Food and water were available ad libitum. The experimental protocols followed the European Community Council Directive and the Animal Care Act (Poland) and were approved by local and national authorities (Local Ethical Committee permission 1456/2023).

Survival surgeries for chronic implantation of the probes (both for histological evaluation and for electrophysiology) were performed under isoflurane anesthesia (5% for induction to 2% for maintenance in oxygen, 0.5 L O_2_ min^−1^) with buprenorphine as a painkiller (subcutaneous injection, 0.1 mg kg^−1^). Post‐operative care included an anti‐inflammatory and analgesic drug (Tolfedine, 2 mg kg^−1^, subcutaneous injection) and an antibiotic (Baytril 5 mg kg^−1^, subcutaneous injection) for 5 days. For consecutive recording sessions (1, 2, and 3 weeks after implantation), mice were sedated with a low dose of ketamine + medetomidine mixture (75 + 0.5 mg kg^−1^, intraperitoneal injection). Non‐survival “acute” electrophysiological experiments were performed under urethane anesthesia (1.5 g kg^−1^ bw, intraperitoneal injection).

In each experiment, fiber‐coated and bare probes were bilaterally implanted, each in one hemisphere, at corresponding left and right coordinates. The fibers were abundantly soaked in a warm PBS before the insertion started. Electrodes were inserted through the small incision in the dura mater to avoid brain dimpling.^[^ ^51^ ^]^ We noted no additional water absorption after the electrode (and its fiber‐coating) touched the brain surface and no increase in tissue resistance. On the contrary, the probe seemed to be penetrating very smoothly within a lubricating medium of the hydrogel coating. Probe shanks for histological evaluation were inserted ≈2 mm posterior to bregma and ≈1 mm lateral from the midline to the depth determined by the length of the probe shaft, i.e., ≈3 mm from the brain surface. The implants were secured with dental acrylics, and the skin was sutured after the surgery was completed. For the electrophysiological recordings, trepan holes were drilled ≈2.5 mm from the midline, and probes were inserted at an angle (≈20°). Electrodes were mounted on Narishige SM‐11 micromanipulator (10 μm/1.2 mm on a knob scale). Insertion was performed manually with the slow progression of the electrode (≈20 μm s^−1^). At a depth of 3.5 mm, the tip of the probe with recording points was within the somatosensory thalamus. Wider placement of electrodes was necessary to ensure enough space for the manipulator holders and head‐stage connectors. The probes were secured to the skull with dental acrylic in the chronic setup. Two small stainless steel screws anchored the headset to the skull. One of them was soldered to a reference pin. Flexible ribbons with connectors were folded and secured within the protective pockets formed from a plastic scaffold and acrylic layer and closed with a “cap” attached by a small neodymium magnet. The whole headset weighed less than 2 g.

##### In Vivo Experiments: Recording and Processing of Electrophysiological Signals

To record the signal from two silicon probes, we used a 512‐channel data acquisition system developed at the AGH University of Science and Technology in Cracow.^[^ ^52^, ^53^ ^]^ The system is based on a dedicated integrated circuit Neurostim‐3 that generates stimulation currents and records neuronal signals at 64 electrodes. Probes with 64 channels Molex connectors (used for acute experiments) were directly attached to compatible AGH head stage. Chronic probes had a connector with two 32‐channel Molex sockets, which required an interface (ADPT‐NZA‐SSB6 from White Matters) to connect them to another AGH head stage. The signal was amplified ×500, filtered (0.6 Hz–9 kHz), and sampled, along with stimuli triggers, at 40 kHz into HDF5 data format.

For evaluation of electrophysiological signals, 5 min continuous data with whisker stimulations were imported to a MATLAB (v 2021b) environment and processed with the use of EEGlab^[^ ^54^ ^]^ function and custom‐written scripts. Signals were low‐pass filtered (EEGlab FIR filter, low‐pass 1000 Hz) and down‐sampled to 4 kHz. Two‐second epochs were extracted around each stimulus marker and averaged. To detect action potentials (spikes), the signal was high‐pass filtered at 200 Hz (EEGlab, cutoff frequency [−6 dB]: 175 Hz, zero‐phase, noncausal). For each electrode contact, the background noise level was estimated as the standard deviation of all data values;^[^ ^55^ ^]^ spikes were detected as peaks exceeding the noise level by a factor of 4 and larger than 20 μV. The former rule ensures that clear spikes are detected in highly active channels;^[^ ^56^ ^]^ the latter rule was set to automatically exclude non‐spike events from low‐activity, bad channels. The mean amplitude of spikes’ peaks divided by a noise level was used as a quantification of the SNR.

We generated a short current pulse at the electrode for the impedance measurement. We measured the resulting electrode voltage using the stimulation and recording circuits integrated into each channel of the Neurostim‐3 chip. The stimulation circuit could generate complex stimulation signals with a current resolution of 8 nA. We used rectangular biphasic pulses with 500 μs per phase duration and ±8 nA amplitude to keep the electrode's voltage low and the current–voltage dependence linear. The electrode voltage amplitude during the measurement was in a few mV ranges for plated electrodes. The recording circuit was set to voltage follower mode to record the electrode voltage, which avoids the band‐pass filtering of the circuit working in default mode (gain 500×). Typically 128 channels were measured in one session. The rectangular pulses were applied to consecutive channels with a time step of 25 ms, so for a given channel, the pulse was repeated every 1.6 s. The electrode voltage waveforms for 400 repetitions were averaged for noise reduction. Visual inspection of the waveform confirmed the capacitive character of the electrode impedance. The capacitance was calculated as *Q*/*V*_amp_, where *Q* = 4 pC and *V*_amp_ was the amplitude of the falling edge of the waveform. The impedance at 1 kHz was calculated based on the capacitance value.

##### In Vivo Experiments: Biocompatibility

Mice were perfused transcardially with 4% paraformaldehyde in buffered saline. Silicone probes were gently removed, and brains were sliced coronally into 40 μm sections using Leica 1850 cryostat. Slices were permeabilized with 0.5% Triton X‐100 and blocked for 4 h in 5% NGS. The following primary antibody dilutions were used: rabbit polyclonal anti‐GFAP, 1:250, rat monoclonal anti‐TNF‐α, 1:200, and mouse monoclonal anti‐Iba‐1, 1:250. All markers were stained on the same slices. Slices were washed 3 × 10 min in PBS and incubated for 2 h at RT with the following secondary antibodies: GFAP, Alexa Fluor 488 goat anti‐rabbit (1:1000), TNF alpha, Alexa Fluor 647 goat anti‐rat (1:1000), Iba‐1, and Alexa Fluor 568 goat anti‐mouse (1:1000). Lastly, preparations were washed in PBS 3 × 10 min and mounted on Superfrost slides in Fluromount with DAPI.

##### In Vivo Experiments: Laser Scanning Microscopy

Sections were imaged using a Zeiss LSM 780 microscope (Carl Zeiss, Jena, Germany) with ZEN software. The following imaging settings were used. DAPI: excitation with a diode laser (*λ*_ex_ = 405 nm), *λ*_em_ = 410–495 nm, BS 405. AF488: excitation with argon laser (*λ*_ex_ = 488 nm), *λ*_em_ = 490–579 nm; AF568: excitation with diode‐pumped solid‐state diode (*λ*_ex_ = 561 nm), *λ*_em_ = 570–650 nm; AF647: excitation with HeNe laser (*λ*_ex_ = 633 nm), *λ*_em_ = 638–755 nm. For all Alexa Fluor probes, BS 488/561/633 was used. Imaging was done over a Plan‐Apochromat 20×/0.8 M27 objective lens with a resolution of 1024 × 1024 pixels. The pinhole was adjusted to 1AU for each fluorochrome. Stacks of images across the slice thickness were obtained with 1 μm z‐increment.

##### In Vivo Experiments: Image Processing and Analysis

Quantitative analysis of the marker‐positive cells was performed using ImageJ. The threshold was estimated using a built‐in algorithm (OTSU). Equal threshold levels were used for each image in the dataset. GFAP, Iba‐1, and TNF‐α levels were estimated by counting the integrated intensity of AF488‐, AF568‐, or AF647‐positive pixels within the image stacks, respectively. Statistical significance was assessed using analysis of variance with post hoc Sidak's multiple comparisons test in Prism software (GraphPad).

## Conflict of Interest

The authors declare no conflict of interest.

## Author Contributions

**Seyed Shahrooz Zargarian**: investigation (equal); methodology (equal); validation (equal); visualization (equal); writing—original draft (equal); and writing—review and editing (supporting). **Chiara Rinoldi**: investigation (equal); methodology (equal); validation (equal); visualization (equal); writing—original draft (equal); and writing—review and editing (supporting). **Yasamin Ziai**: investigation (supporting); methodology (supporting); and writing—review and editing (supporting). **Anna Zakrzewska**: investigation (supporting); methodology (supporting); and writing—review and editing (supporting). **Roberto Fiorelli**: investigation (supporting); methodology (supporting); and writing—review and editing (supporting). **Małgorzata Gazińska**: investigation (supporting); methodology (supporting); and writing—review and editing (supporting). **Martina Marinelli**: investigation (supporting); methodology (supporting); and writing—review and editing (supporting). **Magdalena Majkowska**: investigation (supporting); methodology (supporting); and writing—review and editing (supporting). **Paweł Hottowy**: investigation (supporting); methodology (supporting); and writing—review and editing (supporting). **Bartosz Mindur**: investigation (supporting); methodology (supporting); and writing—review and editing (supporting). **Rafał Czajkowski**: investigation (supporting); methodology (supporting); and writing—review and editing (supporting). **Ewa Kublik**: investigation (supporting); methodology (supporting); writing—review and editing (supporting). **Paweł Nakielski**: investigation (supporting); methodology (supporting); and writing—review and editing (supporting). **Massimiliano Lanzi**: investigation (supporting); methodology (supporting); and writing—review and editing (supporting). **Leszek Kaczmarek**: investigation (supporting); methodology (supporting); and writing—review and editing (supporting). **Filippo Pierini**: conceptualization (lead); funding acquisition (lead); supervision (lead); and writing—review and editing (lead).

## Supporting information

Supplementary Material

## Data Availability

The data that support the findings of this study are available from the corresponding author upon reasonable request.
