# Supplementary material for: Chronic Probing of Deep Brain Neuronal Activity Using Nanofibrous Smart Conducting Hydrogel‐Based Brain–Machine Interface Probes
Source: Small Sci. 2025 Jan 28;5(5):2400463. doi: 10.1002/smsc.202400463 (PMC12087770; doi:10.1002/smsc.202400463)
Supplement: Supplementary file 1 — Supplementary Material [file SMSC-5-2400463-s001.zip › smsc202400463-sup-0001-SuppData-S1.pdf]

Supporting Information

Chronic Probing of Deep Brain Neuronal Activity Using Nanofibrous Smart Conducting Hydrogel-Based Brain-Machine Interface Probes

Seyed Shahrooz Zargarian[1], Chiara Rinoldi[1], Yasamin Ziai[1], Anna Zakrzewska[1], Roberto Fiorelli[1], Małgorzata Gazińska[2], Martina Marinelli[3], Magdalena Majkowska[4], Paweł Hottowy[5], Bartosz Mindur[5], Rafał Czajkowski[4], Ewa Kublik[4], Paweł Nakielski[1], Massimiliano Lanzi[3], Leszek Kaczmarek[4] and Filippo Pierini[1*]

Seyed Shahrooz Zargarian (shzargar@ippt.pan.pl), Chiara Rinoldi (crinoldi@ippt.pan.pl), Yasamin Ziai (yziai@ippt.pan.pl), Anna Zakrzewska (azakrzew@ippt.pan.pl), Roberto Fiorelli (fiorelli.ro@gmail.com), Paweł Nakielski (pnakiel@ippt.pan.pl), Filippo Pierini (fpierini@ippt.pan.pl)

Department of Biosystems and Soft Matter, Institute of Fundamental Technological Research, Polish Academy of Sciences, Warsaw 02-106, Poland

Małgorzata Gazińska (malgorzata.gazinska@pwr.edu.pl)

Department of Engineering and Technology of Polymers, Faculty of Chemistry, Wrocław University of Science and Technology, Wyb. Wyspiańskiego 27, Wroclaw 50-370, Poland

Martina Marinelli (martina.marinelli5@unibo.it), Massimiliano Lanzi (massimiliano.lanzi@unibo.it)

Department of Industrial Chemistry "Toso Montanari", Alma Mater Studiorum University of Bologna, Bologna 40136, Italy

Magdalena Majkowska (m.majkowska@nencki.edu.pl), Rafał Czajkowski (r.czajkowski@nencki.edu.pl), Ewa Kublik (e.kublik@nencki.edu.pl), Leszek Kaczmarek (l.kaczmarek@nencki.edu.pl)

Nencki Institute of Experimental Biology Polish Academy of Sciences, Warsaw 02-093, Poland

Paweł Hottowy (hottowy@agh.edu.pl), Bartosz Mindur (mindur@agh.edu.pl)

Faculty of Physics and Applied Computer Science, AGH University of Krakow, Krakow 30-059, Poland

* Corresponding author: Filippo Pierini (fpierini@ippt.pan.pl)

Contents:

Supplementary notes Note 1- Note 7.

Supplementary figures S1- S16.

Supplementary tables Table S1, Table S2, and Table S3.

Supplementary videos Sup.vid.1 and Sup.vid.2

Supplementary references

Supplementary Notes

Note 1

Although the reactions based on the azobisisobutyronitrile (AIBN) system for the polymerization of *N*-isopropylacrylamide (NIPAM) benefit from a high conversion rate, the hazardous nature of this initiator, the harsh solvents accompanying it, and remnants of this molecule in the final product trigger the need to switch towards greener systems. For instance, ammonium persulfate (APS) initiated free radical polymerization in an aqueous media, which is considered a safer alternative and can alleviate many of the mentioned shortcomings. While the reaction of NIPAM with epoxy-bearing substances in aqueous media has been studied,[1–3] it should be noted that using a redox system based on APS and *N,N,N′,N′*-tetramethylethylenediamine (TEMED) for a set of pure NIPAM and glycidyl methacrylate (GMA) monomers has not been practiced before. In the mentioned system, TEMED accelerates the polymerization reaction of acrylate monomers and increases their conversion rate. Furthermore, the lifetime and yield of free radicals dramatically increase in the presence of TEMED.

Note 2

All the synthesized copolymers (P(NIPAM-*co*-GMA)) were characterized by ^{1}H-NMR spectroscopy in DMSO-d_6 to evaluate their chemical structure and purity degree. The spectra as well as the chemical shifts and assignments, are shown in Figure S5 and Figure S6 (1-3).

In addition to displaying the characteristic singlet at 1.23 ppm, ascribable to the methylenic protons of methacrylate monomer containing the epoxy group (GMA), the spectra for all copolymers clearly show an overall broadening of signals. It is, therefore, possible to confirm the successful copolymerization of the two monomers.

In particular, the molar content of GMA can also be determined by the integral ratio of the signals centered at 1.23 ppm (GMA) and 1.04 ppm (methylenic protons of the isopropyl group of NIPAM block). The calculated molar ratio for all three copolymers is reported in Table S2 and compared to the initial feed. These findings comply with the expected structure of the synthesized material since the initial GMA molar content is substantially retained, especially for NG97.

Moreover, it is also possible to confirm the integrity of the epoxy group of the GMA monomer for all copolymers. Indeed, all spectra clearly show the broad signal at 3.23 ppm, as well as 2.80

and 2.66 ppm, ascribable to the methylenic protons of the epoxy ring and which are only present when the group hydrolysis is prevented.

Note 3

In general, GMA monomers, such as *N*-vinylpyrrolidone and NIPAM, are more reactive than vinyl or acrylate monomers.[4,5] However, as the amount of GMA increases in the feed, the termination probability of the active oligomers becomes the competing factor. Considering that GMA oligomers have low miscibility in water, the propagating poly(glycidyl methacrylate) (PGMA) becomes the active immiscible component in the reaction. If terminated, these GMA-rich macromolecules can egress from the reaction and decrease the GMA content in the NG95 copolymer. In the first steps of the reaction, we observed a brief period in which the NG95 medium became turbid. This heterogenicity points to the formation of GMA-rich oligomers. Since transparency was reached once more, we can consider the extent of termination between hydrophobic active chains is low. The gap between the actual and initial GMA molar contents becomes even wider for NG93, demonstrating the accelerated precipitation of terminated GMA-rich oligomers. We decided to follow this trend for NG90 and NG75; However, GMA phase separation became so persistent that the precipitated agglomerates were visible in the reaction medium. Moreover, the turbidity period was never resolved for these two copolymers. Hence, measuring the molar content of GMA for NG90 and NG75 was challenging and prone to error. The lower reactivity of NIPAM also plays a role during the free radical prorogation. The higher the amount of NIPAM in the feed, the more chains are being initiated, and consequently, the monomer content in the synthesized copolymer decreases. Therefore, this justifies the trend observed in Figure 3c. Moreover, the absence of a second peak in the GPC curves shows that the phase-separated GMA chains were able to escape the dialysis bag.

Note 4

The reaction between the building blocks of GMA and the cross-linker resulted in the formation of a 1:1 adduct, leading to the disappearance of the characteristic epoxide peak, as shown in Figure S8. In this figure, the left arrow highlights the shift of the peak at 1550 cm^{-1}, corresponding to the N-H stretching of the amide group, to higher frequencies in both as-spun and cross-linked samples. This shift can be attributed to the overlap between the characteristic peak of the cross-linkers functional groups and that of the type 2 amine of PNIPAM. Furthermore, the right arrow indicates a shift of the GMA's ester configuration peak to lower

frequencies, resulting from hydrogen bonds between carboxyl groups and the hydroxyl group of the copolymeric chains.

Note 5

The Tg of PNIPAM is typically within the range of 115-148 °C, depending on molecular weight and tacticity.[6] This high Tg makes it unlikely for significant polymeric chain rearrangement to occur during the relatively low-temperature sublimation process, as shown in Figures 4b and 4c.

Note 6

Rapid water infiltration is instrumental in achieving instant shape recovery observed in Sup.vid.1 and Sup.vid.2. When exposed to water, the nanofibrous hydrogel quickly absorbs moisture, leading to rapid and extensive swelling. This swelling process, which is facilitated by the high surface area-to-volume ratio of nanofibers, enables the hydrogel to regain its original shape almost instantaneously. Furthermore, the rapid penetration of water molecules into the nanofibrous network allows the hydrogel to reach its swelling equilibrium quickly. Within a mere 5 seconds, the hydrogel attains its maximum level of swelling, highlighting the efficiency and effectiveness of this nanofibrous hydrogel in responding to changes in its environment. In other words, the initiation of the anticipated shape by water molecules for the fibrous hydrogels of NG95 and NG95PT10 can be regarded as instantaneous.

Note 7:

In deep brain probing, the mechanical strength and precision required for accurate probe insertion and long-term stability are critical.[7] Fully rigid neural probes have reached a state of technological maturity, offering reliable performance but facing limitations in biocompatibility.[8,9] Fully soft probes, while promising in reducing tissue damage, lack the structural integrity necessary for deep brain insertion without the aid of additional, often invasive, tools.[7,10,11] The hybrid approach, interfacing a rigid core with a soft, conductive nanostructured hydrogel coating, addresses these challenges by providing the mechanical robustness needed for deep brain applications while enhancing biocompatibility and signal transmission. This strategy offers a balanced solution that leverages the strengths of both technologies, making it particularly well-suited for chronic deep brain probing.

Supplementary Tables

Table S1. Structural parameters of PT.

Low-angle diffractions (2θ) (°)	High-angle diffractions (2θ) (°)	On-plane Th chain distances (Å)	Plane stacking distances (Å)	Crystallite mean sizes (L) (nm)
5.45; 11.03; 16.84	21.80	16.21	4.07	15.95

Table S2. Comparison of the obtained and theoretical molar ratio of NIPAM and GMA monomers in the copolymers.

**Sample code**	**Composition (initial feed) (%)**		**Composition (final) (%)**	
	**NIPAM**	**GMA**	**NIPAM**	**GMA**
**NG97**	97	3	97.4	2.6
**NG95**	95	5	96.8	3.2
**NG93**	93	7	95.5	4.5

Table S3. Comparative analysis of developed neural probes and key findings.

**Probe type**	**Design approach**	**Probed region**	**Duration of probing**	**Remarks**	**Reference**
**Multifunctional fiber probe**	Fully soft	Various cortical regions	Chronic (months)	Demonstrated Long-term stable *in vivo* recordings, optogenetic stimulation, and drug perturbation for periods over 2 months.	[12]
**Double-layer flexible probe**	Fully soft	Somatosensory cortex	Acute	As the probes can illuminate brain tissue from both sides, the recording electrodes are also cofabricated, and record neuronal activity bi-directionally.	[13]
**3D Macroporous Nanoelectronic Probe**	Fully soft	Hippocampus	Chronic (months)	3D macroporous structure mimicked brain tissue mechanics, reducing immune response and maintaining stable recordings over time.	[14]
**Injectable mesh electronics**	Fully soft	Cortical regions	Chronic (months)	Syringe-injectable mesh electronics allowed for minimally invasive delivery and stable integration with neural tissue.	[15]

**Magnetothermal probe**	Rigid	Deep brain regions	Acute	Wireless magnetothermal stimulation enabled deep brain modulation without implanted wires.	16
**Optetrode**	Rigid	Medial entorhinal cortex	Acute	Enabled single-neuron resolution recording of cortical-projecting MEC layer in freely moving mice.	17
**Foldable electrode array**	Soft	Visual cortex	Acute	A flexible neural probe with active multiplexing was designed. The probe demonstrated high-resolution neural recordings over large cortical areas *in vivo* and potential for mapping brain activity with high spatial resolution.	18
**Flexible neural probe**	Soft	-	Chronic	A fish-bone-shaped flexible neural probe from polyimide was strengthened by biodegradable silk coating for enhanced biocompatibility.<br><br>The silk coating provided temporary stiffness for insertion, which degrades over time to enhance flexibility and biocompatibility.	19
**3D Modular microelectrode array**	Hybrid (rigid with flexible coating)	Non-human motor cortex	Chronic	Demonstrated minimal tissue damage, in accordance with the high quality and stability of the recorded neural activity. Geometry allows both semi-chronic and chronic applications.	20
**Coated BMI's Probe**	Hybrid (rigid with soft coating)	Posterior to Bregma Point	Acute and Chronic (6 weeks)	A rigid BMI probe is coated with conductive semi-IPN nanofibrous hydrogel. The smart coating exhibited rapid swelling, fast shape recovery and enhanced electrical performance at physiological temperatures. The coated probes exhibited lower impedance and higher signal quality compared to bare probes. No significant inflammation was observed in acute tests.	This work

Supplementary Figures

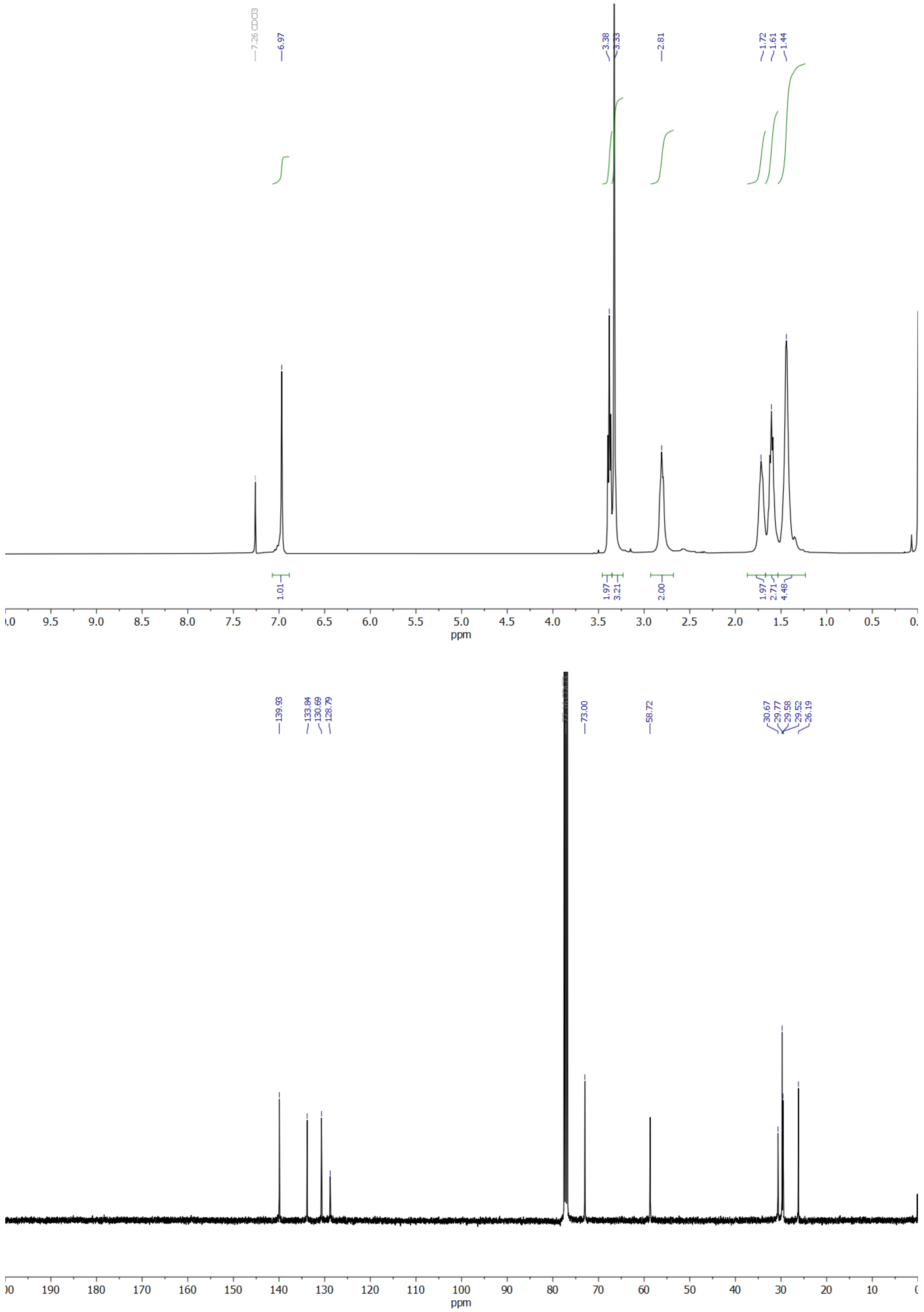

Figure S1. ^{1}H-NMR (top) and ^{13}C-NMR (bottom) spectra of PT6OMe in $CDCl_3$.

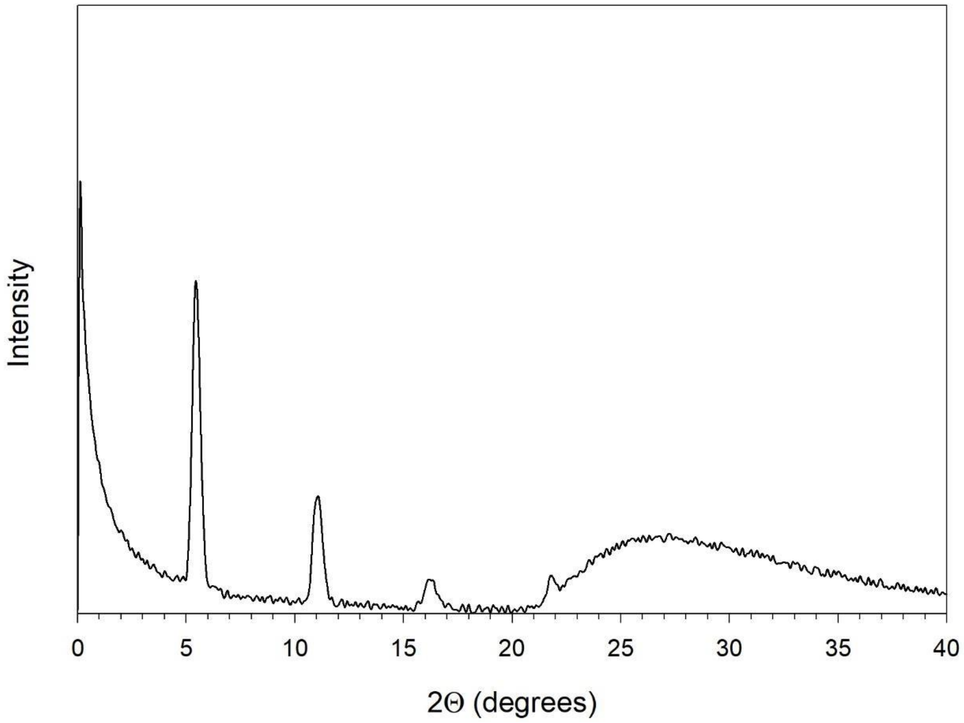

Figure S2. X-ray diffractograms of PT6OMe in film.

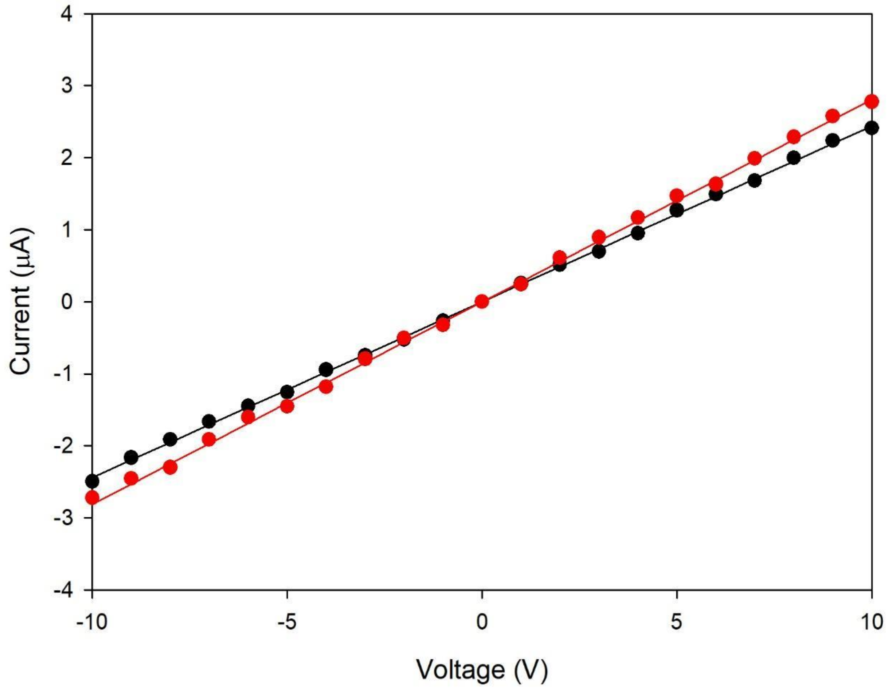

Figure. S3. Current-Voltage characteristics of PT6OMe in film measured in vacuum (black) and air (red). Lines: fitting curves, dots: real values.

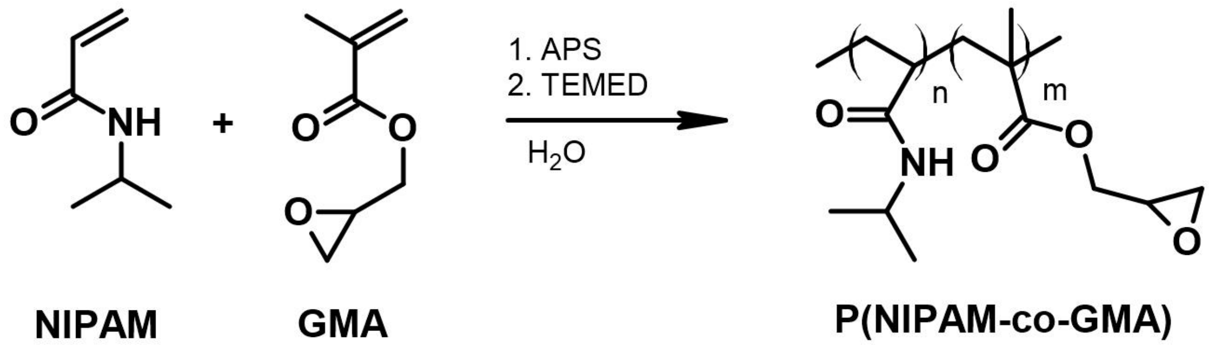

Figure S4: Schematic of the P(NIPAM-*co*-GMA) copolymerization reaction.

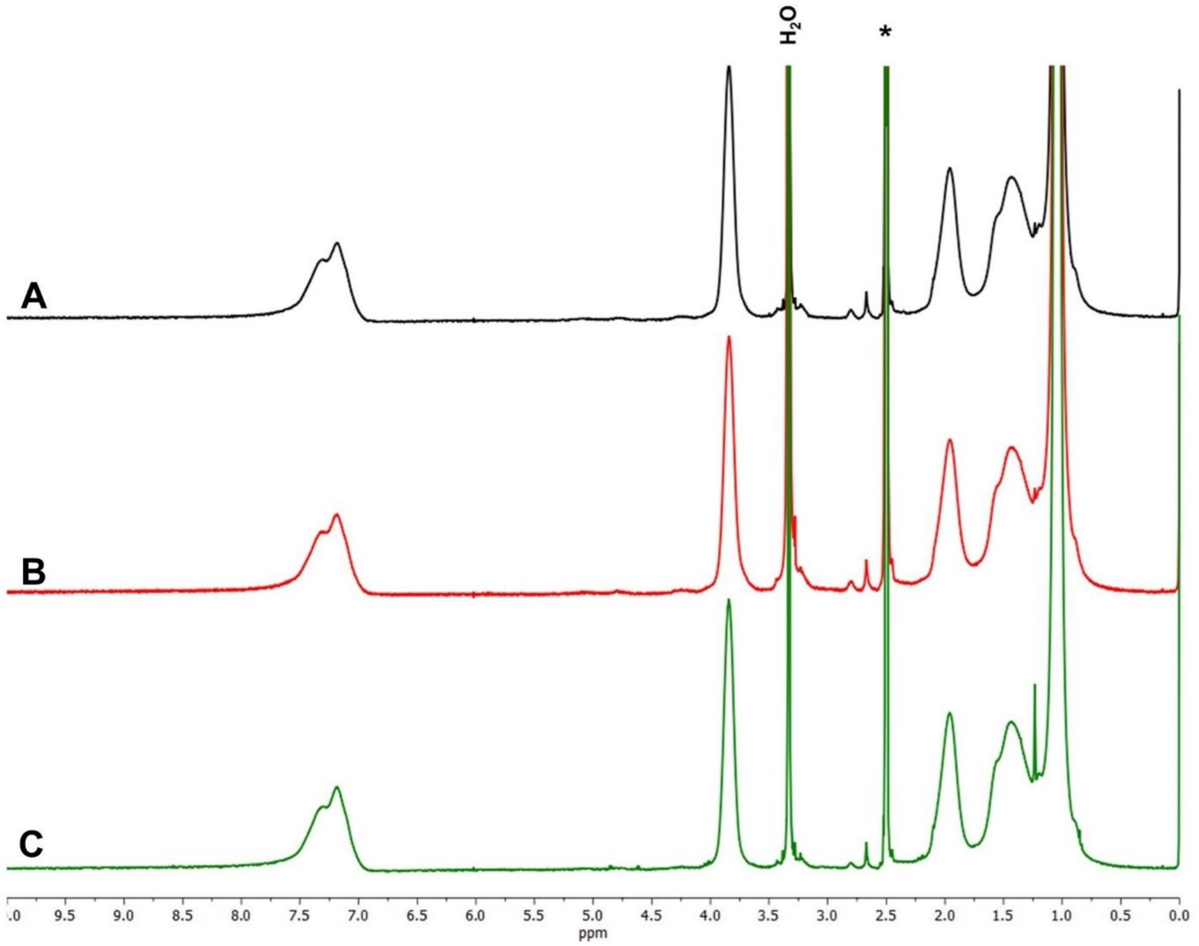

Figure S5. ^{1}H-NMR spectra of copolymers (A) NG97, (B) NG95 and (C) NG93. Asterisk: solvent resonance (DMSO-d_6).

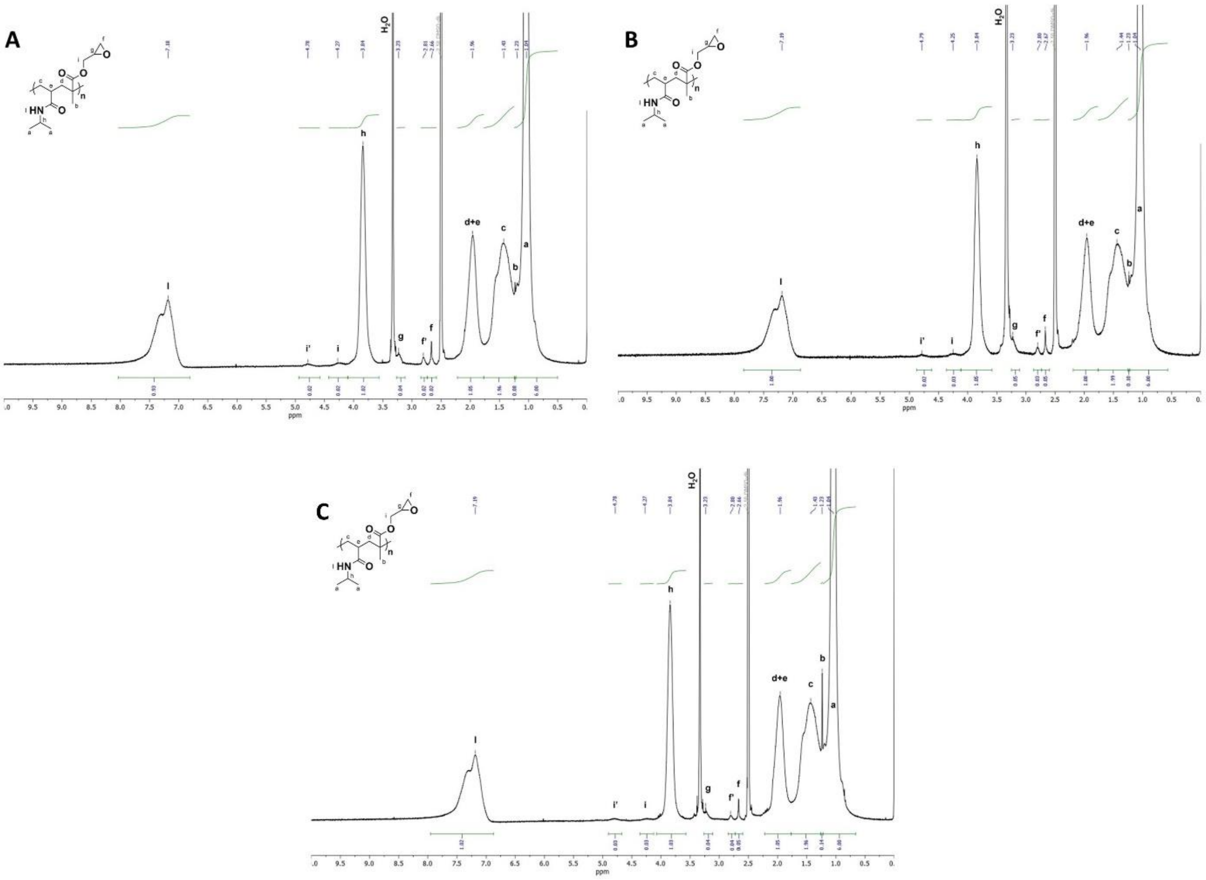

Figure S6. ^{1}H-NMR spectrum of (A) NG97, (B) NG95, and (C) NG93 in DMSO-d_6.

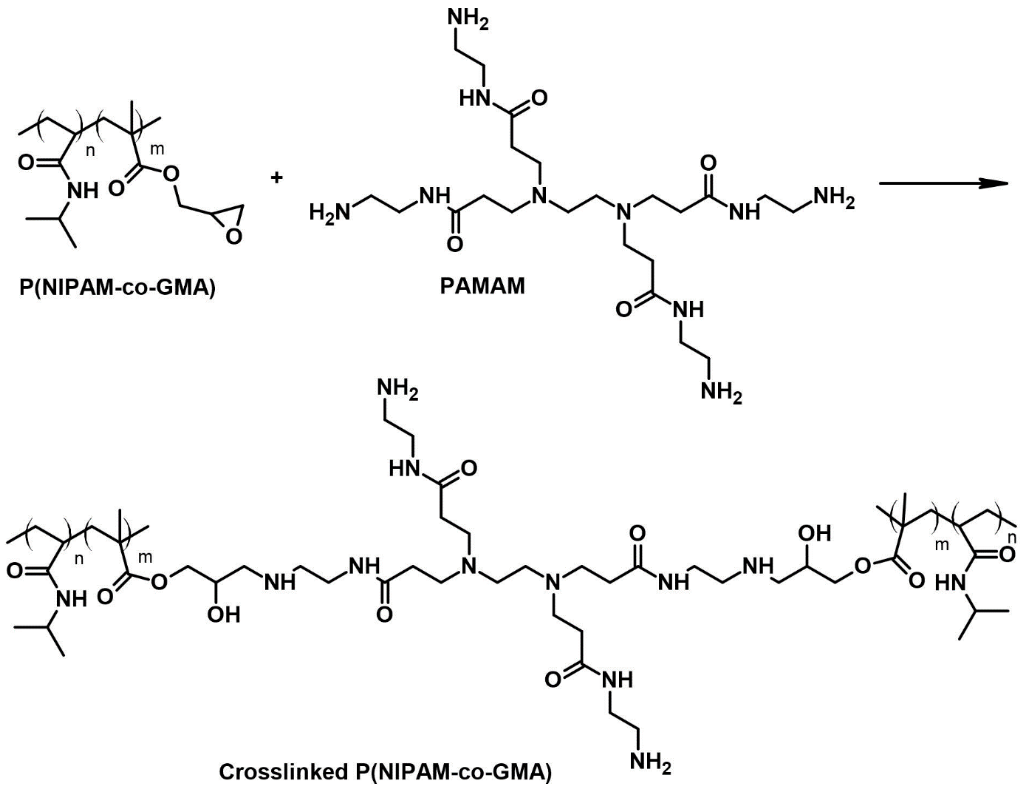

Figure S7: Schematic of the P(NIPAM-*co*-GMA) copolymerization reaction.

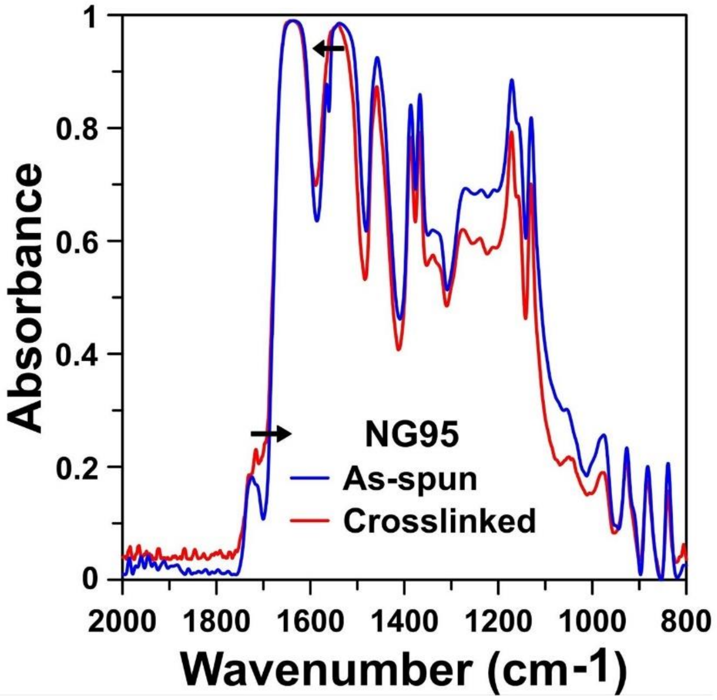

Figure S8. FT-IR spectra of the as-spun and cross-linked NG95 electrospun fibers. The left arrow indicates the shift of the peak at 1550 cm^{-1}, assigned to the N-H stretching of the amide group, to higher frequencies in as-spun and heat-treated samples due to an overlap between the characteristic peak of the

crosslinker functional groups with that of type 2 amide of P(NIPAAm). The right arrow shows a shift of the GMA's ester configuration peak to lower frequencies due to the formation of hydrogen bonding between carboxyl groups and the hydroxyl group of the copolymeric chains.

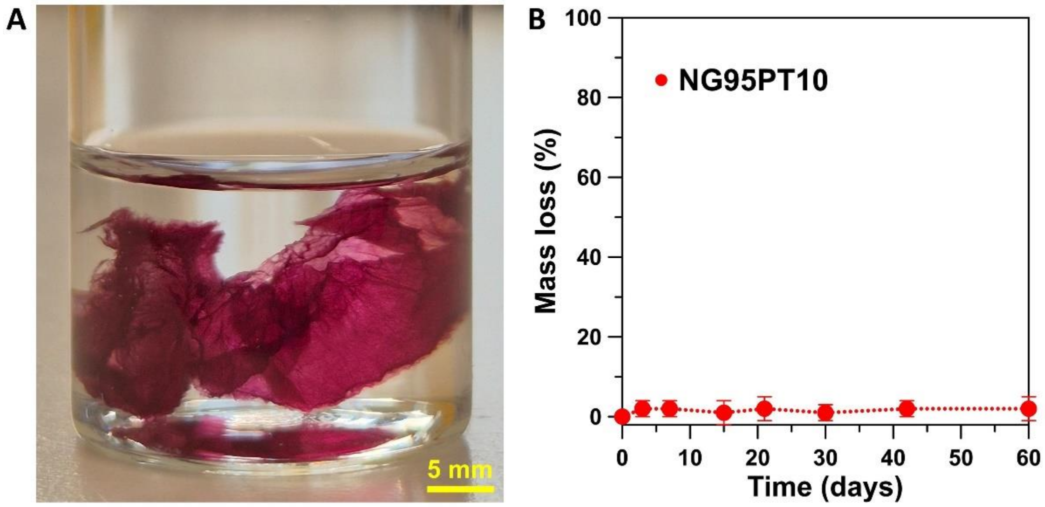

Figure S9. *In vitro* stability of the conductive semi-IPN NG95PT10 nanofibrous hydrogel. (A) Camera photo showing a piece of NG95PT10 nanofibrous hydrogel immersed in PBS for 12 months. The hydrogel fabric remained perfectly intact, unaltered, and with no residues of PT detectable in PBS. (B) Mass loss measurements of NG95PT10 nanofibrous hydrogel in PBS over two months.

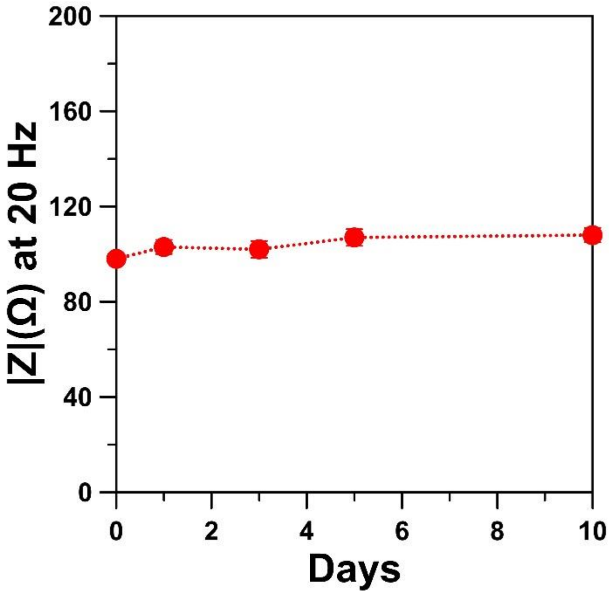

Figure S10. Demonstration of electrochemical stability of NG95PT10 semi-IPN fibrous hydrogel. Impedance values at 20 Hz were measured for hydrated NG95PT10 circular specimens over 10 days. The data indicate stable impedance throughout the tested period.

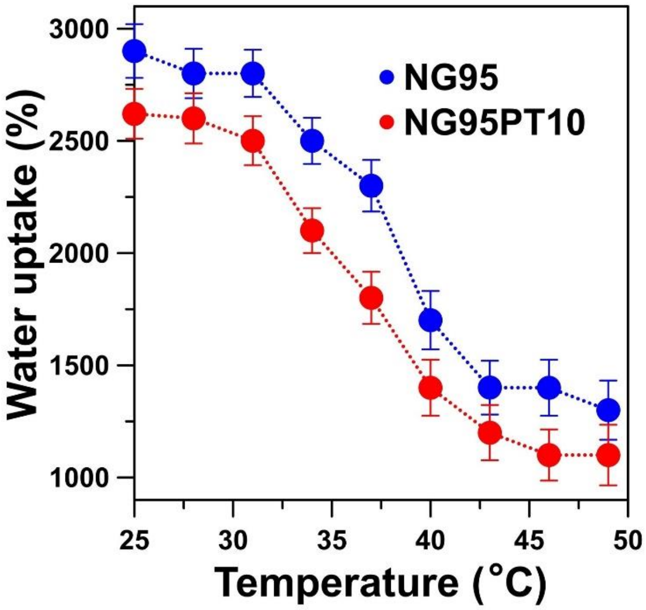

Figure S11. Swelling behavior of NG95 and NG95PT10 fibrous hydrogels at varying temperatures. At room temperature (23 °C), both hydrogels exhibit excellent water uptake. As the temperature approaches the VPT temperature of 33 °C, syneresis begins, leading to a gradual decrease in water uptake. At body temperature (37 °C), NG95PT10 retains approximately 18 times its dry weight in water.

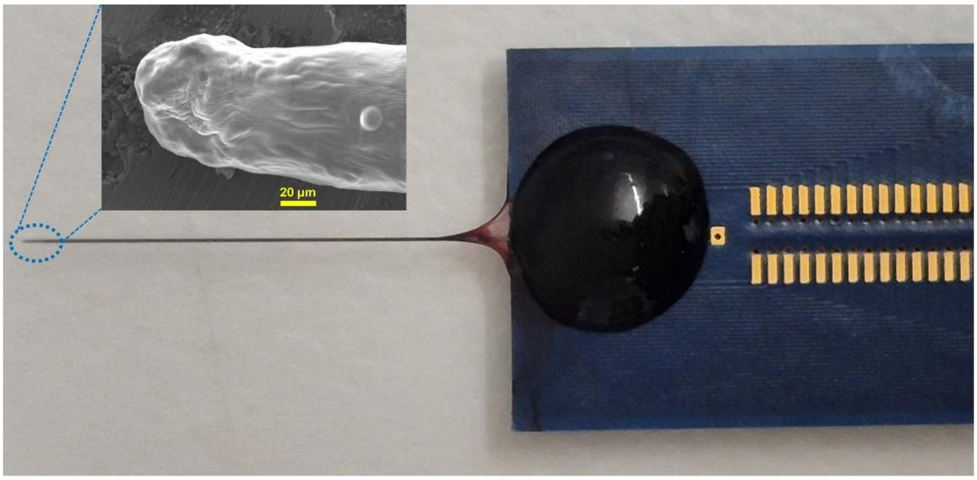

Figure S12. Post-implantation condition of the NG95PT10-coated BMI probe after retraction from the mice brain. A camera photo of the BMI probe shows the coated prong tip and the encapsulated wire bonds located above the printed circuit. The inset SEM image of the prong tip shows the NG95PT10 fibrous hydrogel coating, which, despite being accumulated with biological residues, retains its fibrous architecture and structural integrity, adhering firmly to the probe after retraction.

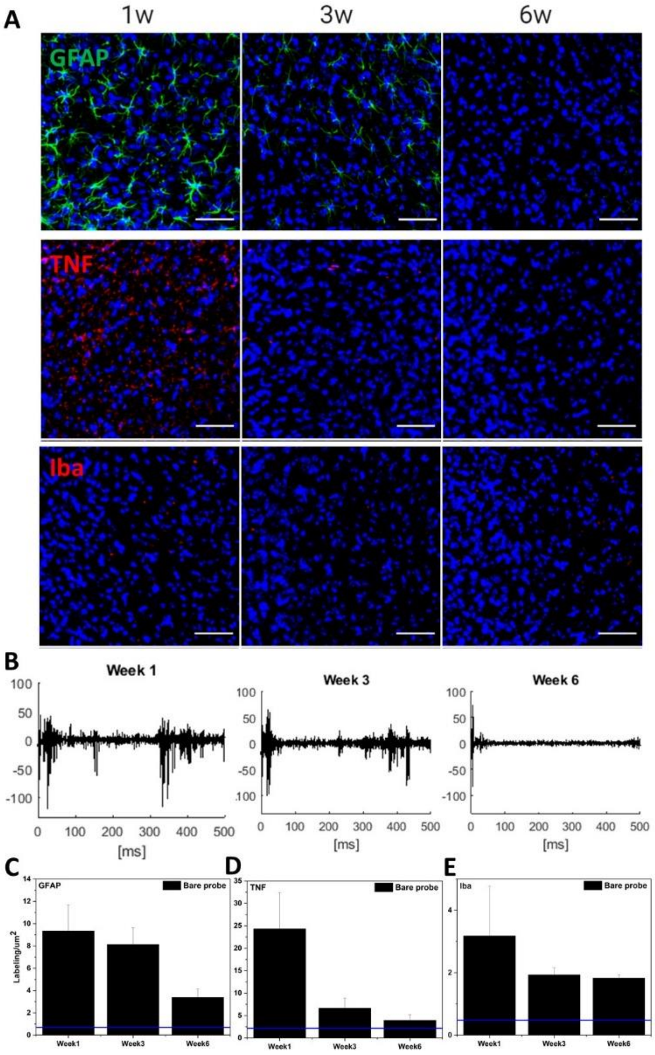

Figure S13. ***In vivo*** **chronic experiments of bare neural probes.** (A) Confocal images of immunostained brain slices after bare probe implantation for up to 6 weeks. Neuroinflammation markers GFAP (green color), Iba-alpha (red color), and TNF-alpha (red color) were immunolabeled. Nuclei were stained with DAPI (blue color). All images are oriented in the same manner. Scale bars: 40 µm. (B) Chronic recording through bare neural probes: brain signal waveforms; (C-E) Fluorescence quantification of (C) GFAP, (D) TNF-alpha, and (E) Iba1, plotted in comparison with untreated conditions (blue line).

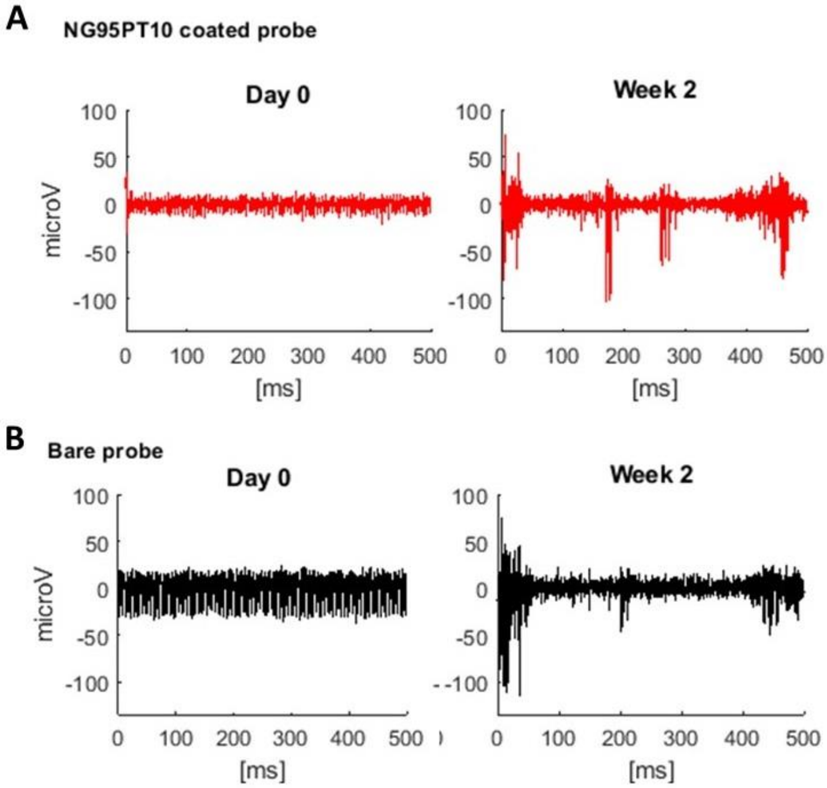

Figure S14. Chronic recording through NG95PT10 coated neural probes (A) *vs.* bare neural probes (B): brain signal waveforms recorded at day 0 and week 2.

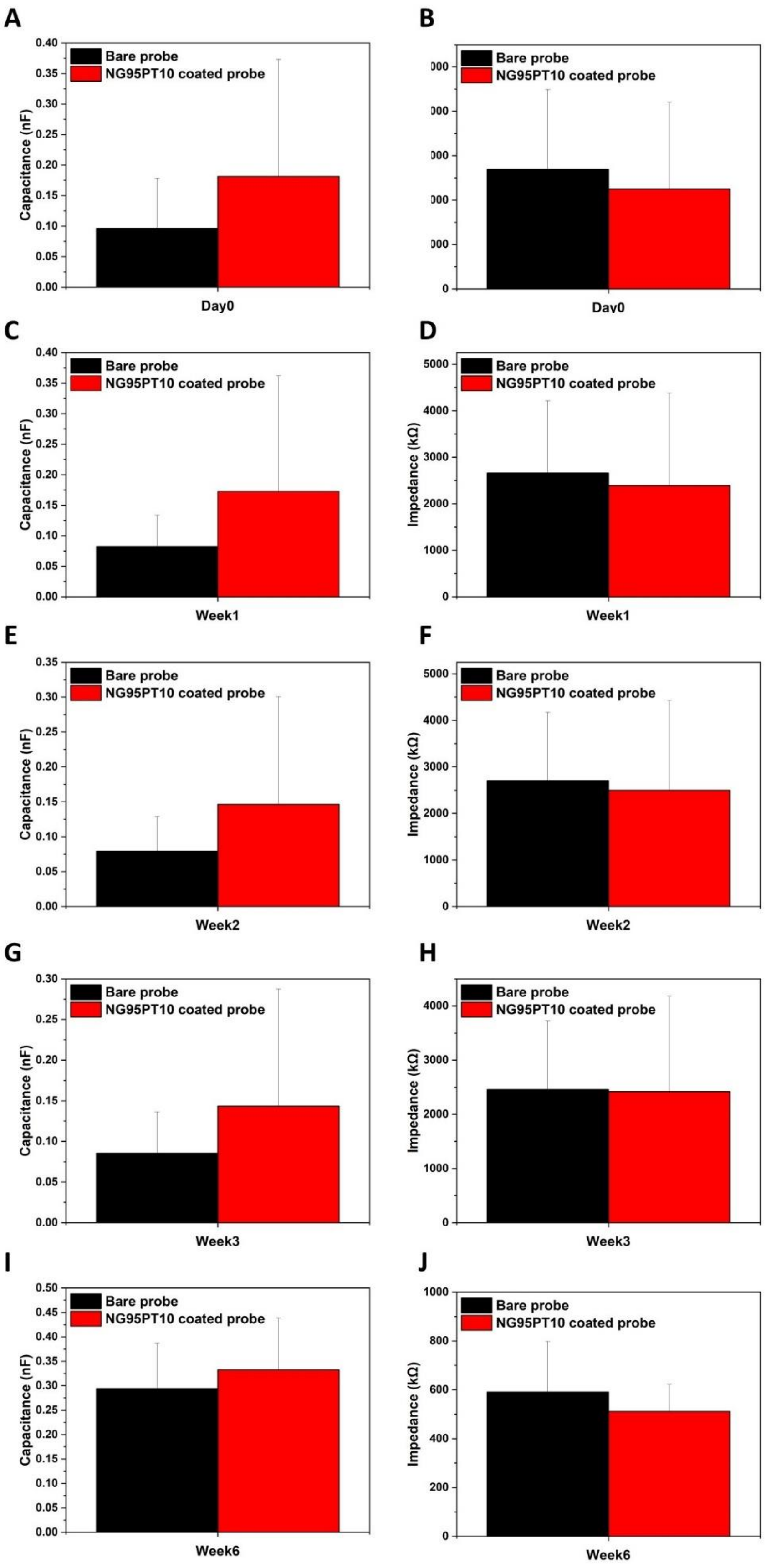

Figure S15. Chronic recording through NG95PT10 coated neural probes *vs.* bare neural probes: (A,C,E,G,I) capacitance and (B,D,F,H,J) impedance.

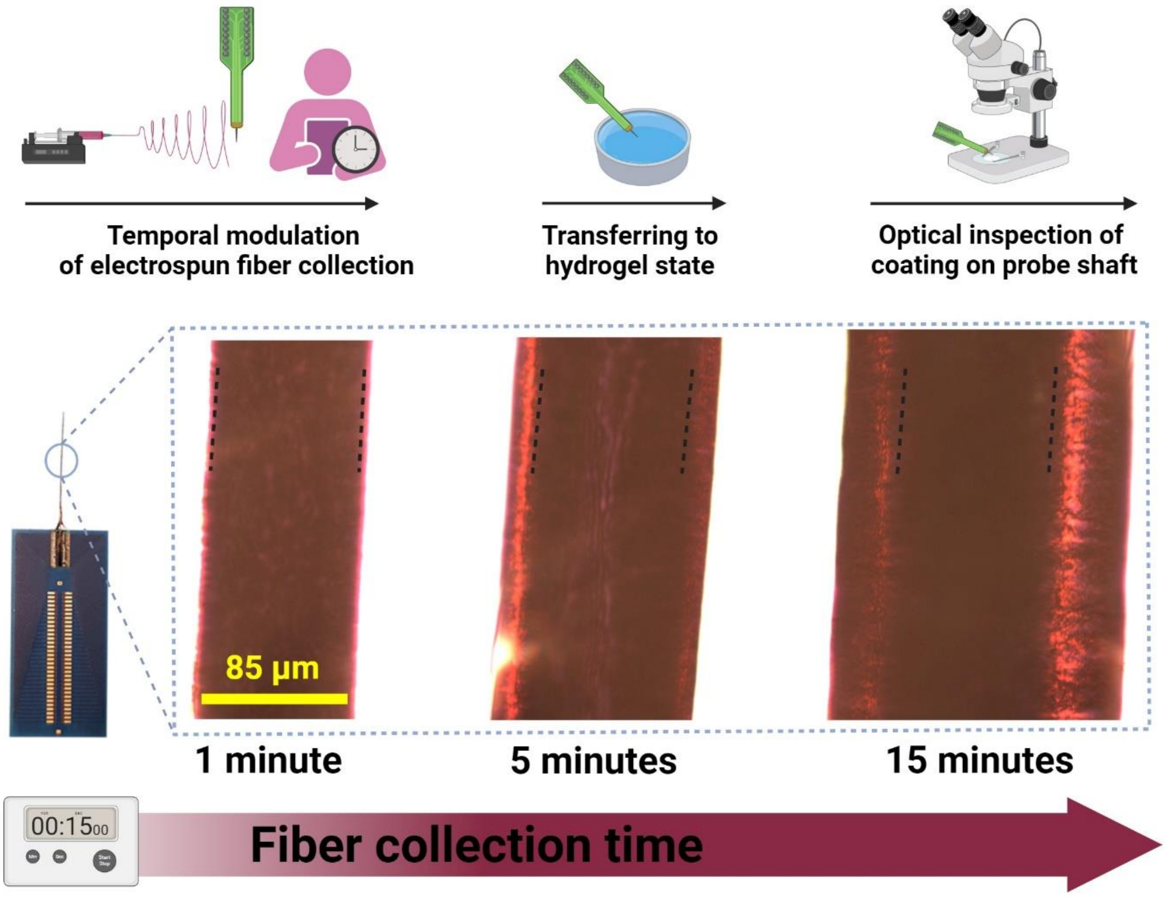

Figure S16. Demonstration of precise control over coating thickness enabled by the electrospinning process. The schematic illustrates the temporal modulation of fiber collection onto neural probe shafts, which are then transformed into a hydrogel state. Light microscope images display the resulting coatings after 1, 5, and 15 minutes of fiber collection, corresponding to coating thicknesses of approximately 3, 15, and 40 µm, respectively, demonstrating the tunability of coating thickness through simple adjustments in fiber collection time. The 5-minute collection time represents the standard used for the *in vivo* studies.

Supplementary Videos

Sup.vid.1: Fast shape recovery of NG95 fibrous hydrogel. The nanostructured hydrogel recovers its shape instantly upon release into water.

Sup.vid.2: Fast shape recovery of the semi-IPN fibrous hydrogel (NG95PT10) showing the same shape recovery behavior as NG95.

Supplementary References

1. Chen, L., Dong, J., Ding, Y. & Han, W. Environmental responses of poly(N-isopropylacrylamide-co-glycidyl methacrylate derivatized dextran) hydrogels. *J. Appl. Polym. Sci.* **96,** 2435–2439 (2005).

2. van Dijk-Wolthuis, W. N. E. *et al.* Synthesis, Characterization, and Polymerization of Glycidyl Methacrylate Derivatized Dextran. *Macromolecules* **28,** 6317–6322 (1995).

3. Reis, A. V. *et al.* Copolymer hydrogel microspheres consisting of modified sulfate chondroitin-co-poly(N-isopropylacrylamide). *J. Appl. Polym. Sci.* **121,** 2726–2733 (2011).

4. Hunter, T. C. & Price, G. J. Glycidyl methacrylate and N-vinylpyrrolidinone copolymers: synthesis and nuclear magnetic resonance characterization. *Polymer* **35,** 3530–3534 (1994).

5. Virtanen, J. & Tenhu, H. Studies on copolymerization ofn-isopropylacrylamide and glycidyl methacrylate. *J. Polym. Sci. A Polym. Chem.* **39,** 3716–3725 (2001).

6. Biswas, C. S. *et al.* Effects of Tacticity and Molecular Weight of Poly(*N* -isopropylacrylamide) on Its Glass Transition Temperature. *Macromolecules* **44,** 5822–5824 (2011).

7. Weltman, A., Yoo, J. & Meng, E. Flexible, penetrating brain probes enabled by advances in polymer microfabrication. *Micromachines (Basel)* **7,** (2016).

8. Zeng, Q. & Huang, Z. Challenges and opportunities of implantable neural interfaces: from material, electrochemical and biological perspectives. *Adv. Funct. Mater.* (2023). doi:10.1002/adfm.202301223

9. Shi, J. & Fang, Y. Flexible and implantable microelectrodes for chronically stable neural interfaces. *Adv. Mater. Weinheim* **31,** e1804895 (2019).

10. Zhu, J. *et al.* Intelligent soft surgical robots for next-generation minimally invasive surgery. *Advanced Intelligent Systems* **3,** 2100011 (2021).

11. Han, C. *et al.* Recent Advances in Sensor-Actuator Hybrid Soft Systems: Core Advantages, Intelligent Applications, and Future Perspectives. *Adv Sci (Weinh)* **10,** e2302775 (2023).

12. Canales, A. *et al.* Multifunctional fibers for simultaneous optical, electrical and chemical interrogation of neural circuits in vivo. *Nat. Biotechnol.* **33,** 277–284 (2015).

13. Reddy, J. W. *et al.* High Density, Double-Sided, Flexible Optoelectronic Neural Probes With Embedded μLEDs. *Front. Neurosci.* **13,** 745 (2019).

14. Xie, C. *et al.* Three-dimensional macroporous nanoelectronic networks as minimally invasive brain probes. *Nat. Mater.* **14,** 1286–1292 (2015).

15. Liu, J. in *Biomimetics Through Nanoelectronics* 65–93 (Springer International Publishing, 2018). doi:10.1007/978-3-319-68609-7_5

16. Chen, R., Romero, G., Christiansen, M. G., Mohr, A. & Anikeeva, P. Wireless magnetothermal deep brain stimulation. *Science* **347,** 1477–1480 (2015).

17. Yang, X. *et al.* Optrode recording of an entorhinal-cortical circuit in freely moving mice. *Biomed. Opt. Express* **14,** 1911–1922 (2023).

18. Viventi, J. *et al.* Flexible, foldable, actively multiplexed, high-density electrode array for mapping brain activity in vivo. *Nat. Neurosci.* **14,** 1599–1605 (2011).

19. Wu, F., Im, M. & Yoon, E. A flexible fish-bone-shaped neural probe strengthened by biodegradable silk coating for enhanced biocompatibility. in *2011 16th International Solid-State Sensors, Actuators and Microsystems Conference* 966–969 (IEEE, 2011). doi:10.1109/TRANSDUCERS.2011.5969356

20. Barz, F. *et al.* Versatile, modular 3D microelectrode arrays for neuronal ensemble recordings: from design to fabrication, assembly, and functional validation in non-human primates. *J. Neural Eng.* **14,** 036010 (2017).
